# Crystalline Antimony Selenide Thin Films for Optoelectronics through Photonic Curing

**DOI:** 10.1021/acs.chemmater.4c00540

**Published:** 2024-06-07

**Authors:** Udari Wijesinghe, William D. Tetlow, Pietro Maiello, Nicole Fleck, Graeme O’Dowd, Neil S. Beattie, Giulia Longo, Oliver S. Hutter

**Affiliations:** † Department of Mathematics, Physics, and Electrical Engineering, Northumbria University, Newcastle upon Tyne NE1 8QH, United Kingdom; ‡ Jaguar Landrover, Banbury Road, Gaydon CV35 0RR, United Kingdom

## Abstract

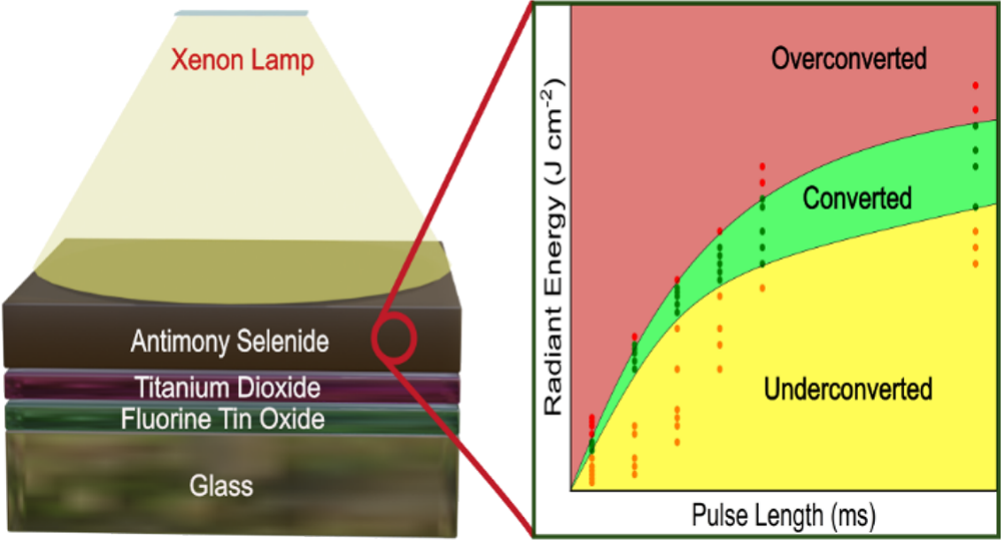

Thermal annealing is the most common postdeposition technique used to crystallize antimony selenide (Sb_2_Se_3_) thin films. However, due to slow processing speeds and a high energy cost, it is incompatible with the upscaling and commercialization of Sb_2_Se_3_ for future photovoltaics. Herein, for the first time, a fast-annealing technique that uses millisecond light pulses to deliver energy to the sample is adapted to cure thermally evaporated Sb_2_Se_3_ films. This study demonstrates how photonic curing (PC) conditions affect the outcome of Sb_2_Se_3_ phase conversion from amorphous to crystalline by evaluating the films’ crystalline, morphological, and optical properties. We show that Sb_2_Se_3_ is readily converted under a variety of different conditions, but the zone where suitable films for optoelectronic applications are obtained is a small region of the parameter space. Sb_2_Se_3_ annealing with short pulses (<3 ms) shows significant damage to the sample, while using longer pulses (>5 ms) and a 4–5 J cm^–2^ radiant energy produces (211)- and (221)-oriented crystalline Sb_2_Se_3_ with minimal to no damage to the sample. A proof-of-concept photonically cured Sb_2_Se_3_ photovoltaic device is demonstrated. PC is a promising annealing method for large-area, high-throughput annealing of Sb_2_Se_3_ with various potential applications in Sb_2_Se_3_ photovoltaics.

## 1 Introduction

Antimony selenide (Sb_2_Se_3_) has emerged as a strong candidate for thin film solar cell applications and water splitting devices with a current power conversion efficiency (PCE) of 10.57% for photovoltaics.^1−3^ Currently, almost all Sb_2_Se_3_ solar cells with high PCE are fabricated by physical vapor deposition at high temperatures (>300 °C) to obtain crystalline Sb_2_Se_3_ with (211)- and (221)-oriented thin films (space group *Pnma* (62)), which are favorable for carrier transport.^4−7^ Postdeposition annealing techniques are often used to improve the absorber quality (i.e., to increase crystallinity, tune nanoribbon orientation, decrease nonradiative recombination loss, and reduce the bulk trap density), which takes 30 to 120 min.^6−9^ Because of this, optimization of production throughput by eliminating rate-limiting equilibrium thermal annealing steps is essential to lower the manufacturing cost of device fabrication. In addition, high-temperature annealing causes mechanical failure in flexible substrates due to the mismatch in thermal expansion coefficient between different layers.^10,11^ Therefore, thermal treatment is undesirable in terms of stability, cost, high volume commercial production, and roll-to-roll manufacturing of such devices. This opens up the need for low-temperature, high-throughput processes to be developed without compromising the Sb_2_Se_3_ quality, which can directly affect the device’s performance.

Recently, laser annealing^12^ was investigated to replace thermal annealing (TA) in Sb_2_Se_3_ solar cell fabrication because of the fast processing speeds and low energy consumption. However, laser-annealed samples were obtained by raster scanning of the laser over the sample, which induces crystal growth along the movement of the laser spot. As such, crystalline regions have an associated directionality, and processing speed is limited by the surface area of the sample. Recently, multiple research groups have reported the use of photonic curing (PC) to anneal perovskite,^13−15^ CuInSe_2_^16^ absorber materials, and metal oxide transport layers, such as SnO_2_,^17,18^ TiO_2_,^19,20^ and NiO.^21,22^ PC enables rapid processing of thin films (10 μs to 100 ms) with intense light pulses from a broadband (200–1500 nm) xenon flash lamp. Varying the voltage across the bulb and changing the duration of the flash pulse control the amount of energy irradiated. In optimizing these parameters, it is therefore possible to achieve the necessary energy to complete the desired annealing in the shortest time. Sb_2_Se_3_ films readily absorb the emitted light, resulting in localized heating and subsequent crystallization. The absorbance difference between the Sb_2_Se_3_ film and the transparent substrate combined with the very short energy delivery time causes non-equilibrium heating. It thus induces a thermal gradient along the material stack, which allows Sb_2_Se_3_ to be selectively heated at a significantly higher temperature, hence enabling the processing of Sb_2_Se_3_ films without heating the substrates. Thus, it eliminates the damage to the underlying layers and maximizes the remaining thermal budget of the device stack.

To date, PC has not been utilized in the crystallization of any antimony chalcogenide materials, and this is the first report for antimony selenide. This work explores the implications of altering the PC parameter window to obtain crystalline Sb_2_Se_3_ films millions of times faster than thermally annealed films (milliseconds compared to minutes) with greater energy efficiency. The resulting Sb_2_Se_3_ films’ structural and optoelectronic properties are reported and understood in terms of energy delivered to the sample and modeled film temperature profiles. This work demonstrates new opportunities in the fabrication of Sb_2_Se_3_ solar cells and has great applicability to other technologies by eliminating the rate-limiting annealing step and making it possible to envision continuous roll-to-roll processes. A proof-of-concept photonically cured Sb_2_Se_3_ photovoltaic device is also demonstrated.

## 2 Experimental Details

### 2.1 Thin Film Fabrication

FTO-coated glass substrates (FTO: TEC15 Sigma-Aldrich) were cleaned using sequential ultrasonic baths of DI water, acetone, and IPA, followed by UV-ozone (Ossila) treatment for 15 min. The cleaned FTO substrates were spin-coated with two doses of 0.30 M titanium isopropoxide solutions in ethanol at 3000 rpm for 30 s and dried at 120 °C for 10 min in the glovebox after each deposition step. The substrates were then annealed in air at 450 °C for 30 min and cooled rapidly to form high-quality titania layers. Sb_2_Se_3_ absorber films were deposited by thermal evaporation (Univex 250 special, Oerlikon Leybold) at a pressure of 1 × 10^–6^ mbar to obtain 1 μm-thick Sb_2_Se_3_ films. Two different TA Sb_2_Se_3_ films were prepared to reference against PC samples: one through annealing at 400 °C for 5 min on a hot plate in nitrogen (TA-N), while another was annealed at 400 °C for 45 min in vacuum (TA-V) in a rapid thermal evaporator (RTE). Sb_2_Se_3_ films were photonically cured using a NovaCentrix PulseForge Invent (500 V/3 A power supply, one capacitor bank capable of delivering 20 J cm^–2^ maximum radiant energy, a 150 mm × 20 mm lamp with 300 mm × 75 mm maximum illumination area). PC was carried out immediately after thermal evaporation. The samples were placed in the sample chamber filled with N_2_, and all PC was performed at ambient temperature (18–20 °C). With the exception of the X-ray photoelectron spectroscopy (XPS) data, all samples were processed with one light pulse; therefore, only the pulse voltage and length are the independent process variables. The radiant energy under each PC processing condition was measured by using a bolometer. The standard deviation in radiant energy for each set of bolometry measurements was less than 1%.

### 2.2 Device Fabrication

All the devices characterized in this work are in superstrate configuration: glass/FTO/TiO_2_/Sb_2_Se_3_/P3HT/Au. The film fabrication procedure with RTE is given in the Supporting Information. Onto the PC-, TA-, or RTE-processed Sb_2_Se_3_ films, a hole transport layer was deposited by spin-coating 4 mg mL^–1^ poly(3-hexylthiophene-2,5-diyl) (P3HT) in chlorobenzene under nitrogen at 1000 rpm for 10 s followed by 4000 rpm for 30 s. Finally, cells were completed by thermally evaporating 100 nm gold through a shadow mask to create cells with an active area of 0.07 cm^–2^.

### 2.3 Characterization

Film thicknesses were measured by using a stylus profilometer (DektakXT, Bruker). X-ray diffraction (XRD) patterns showing the crystal structure and growth nature of the Sb_2_Se_3_ films were recorded using a Rigaku SmartLab SE, in Bragg–Brentano geometry with Cu Kα radiation (1.5418 Å). Surface and cross-sectional morphologies were determined by scanning electron microscopy (SEM; TESCAN MIRA 3) at 5 kV. Elemental compositional analysis was performed using energy-dispersive X-ray spectroscopy (EDX; Oxford Instruments X-max 150) coupled to SEM with an electron acceleration voltage of 10 kV for quantitative acquisitions. XPS data were collected using a Kratos Axis Supra+ with a monochromated Al Kα X-ray source (*h*ν = 1486.6 eV) operating at 450 W and a hemispherical electron energy analyzer operating with a constant pass energy of 20 eV. The sample emission current was 20 mA, and sample charging was neutralized with low energy electrons. In analysis, the data were charge-corrected using the C 1s peak at 284.8 eV. The energy resolution was determined to be 0.4 eV from fitting a Gaussian-broadened Fermi–Dirac distribution to the Fermi edge of a polycrystalline silver reference sample, allowing binding energy determination with a precision of ±0.1 eV. The surface roughness of the films was calculated from the surface topography images taken by atomic force microscopy (AFM; Veeco Dimension 3100). Transmittance and reflectance spectra were measured by using a UV–vis spectrometer (Shimadzu UV-2600 plus). Raman spectroscopy was carried out using a Horiba Jobin Yvon LabRam 300 confocal microscope equipped with a He–Ne laser (λ_exc_ = 633 nm) and a Peltier-cooled CCD detector. The power of the laser at the point of confocality with a 10× objective was 1.36 mW. All of the measurements were carried out at room temperature. The peak positions were calibrated to the 521 cm^–1^ peak of the reference silicon sample. Current density–voltage measurements were conducted under an AM 1.5G solar simulator (Abet Technologies sun 2000) calibrated with a c-Si reference solar cell using a 2400 Keithley source meter.

### 2.4 Simulation

The temperature profile (film temperature versus time) was simulated using SimPulse software by NovaCentrix for a single photonic pulse. The sample stack is modeled as (from the bottom up) 2.2 mm soda lime glass, 360 nm FTO, 90 nm TiO_2_, and 1 μm Sb_2_Se_3_. Material properties of the glass, FTO, and TiO_2_ are based on the SimPulse database, and full details are given in Table S1. The SimPulse model assumes that the light pulse is absorbed on the sample’s surface, and the heat generated there is either conducted down through the sample stack or removed from the sample via convective heat transfer to air from the top and bottom surfaces.^23,24^ Therefore, any photochemical processes that may occur during PC are not taken into account. The sample is assumed to have air above and below it, and the boundary conditions model a convective heat transfer coefficient of 15 W m^–2^ K^–1^ on the top and bottom sample surfaces.

## 3 Results and Discussion

**Figure 1:**
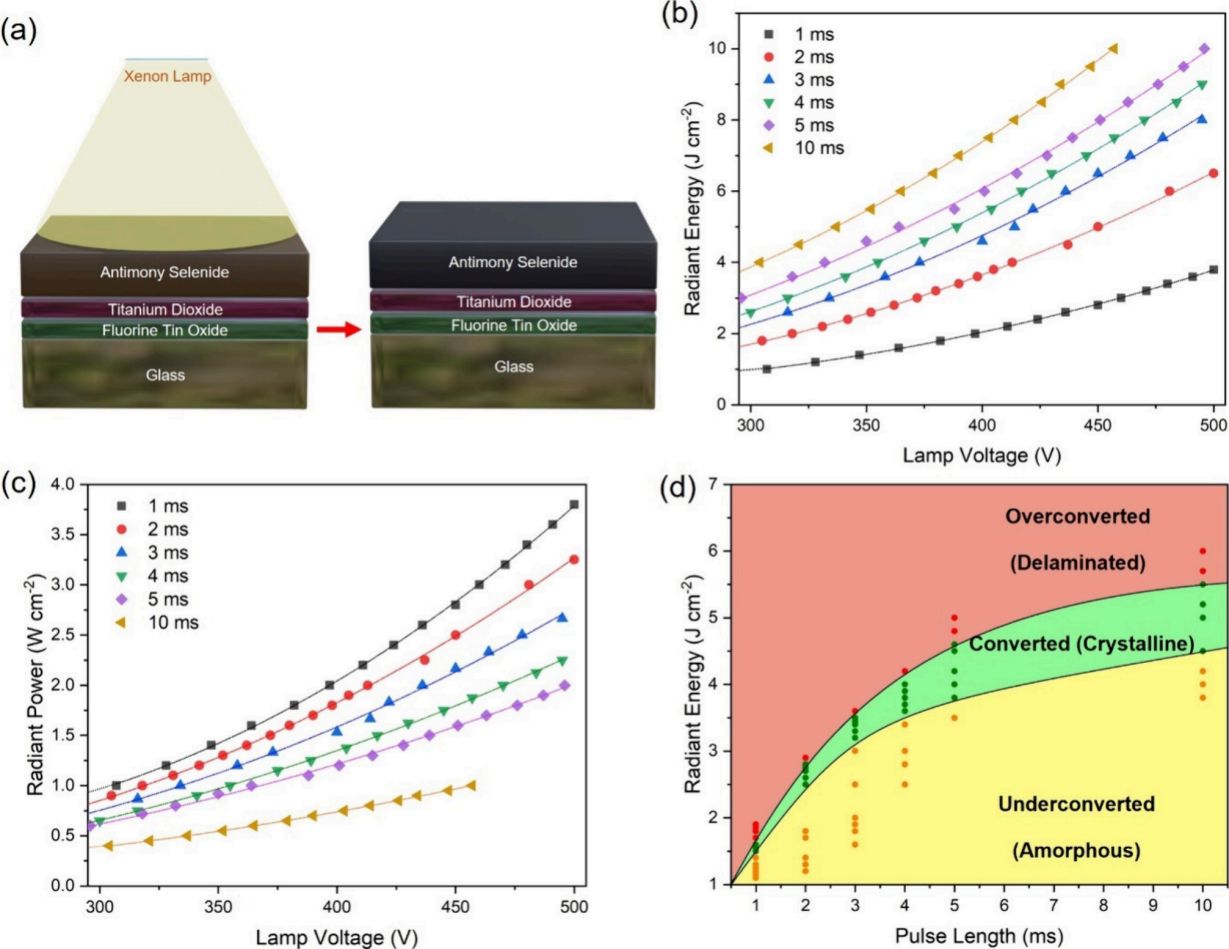
(a) Schematic of the PC process of the Sb_2_Se_3_ film on the glass/FTO/TiO_2_ substrate. (b) Radiant energy as a function of lamp voltage for different pulse lengths measured by a bolometer. (c) Radiant power vs lamp voltage for different pulse lengths. (d) Summary of photonic curing outcomes as a function of radiant energy and pulse length.

A schematic representation of the PC process used in this study is shown in Figure 1a. The PC process includes many processing variables, such as the lamp voltage, pulse length, number of pulses, and pulse rate. Even if the lamp voltage can be controlled directly, the lamp intensity depends on the voltage, lamp size and type, and system configuration. Therefore, a more physically meaningful quantity is the radiant energy delivered to a sample. In this study, we use single pulse curing of Sb_2_Se_3_ such that the two independent variables are radiant exposure, i.e., total radiant energy delivered to the sample by a single light pulse per unit area (J cm^–2^), and pulse length, i.e., duration of a single light pulse (ms). Figure 1b shows the dependence of radiant energy on the lamp voltage for different pulse lengths measured from the bolometer for the PC setup in this study. The calculated average radiant power (W cm^–2^) versus lamp voltage is indicated in Figure 1c (the assumption made in the average radiant power calculation is discussed in the Supporting Information).

**Figure 2:**
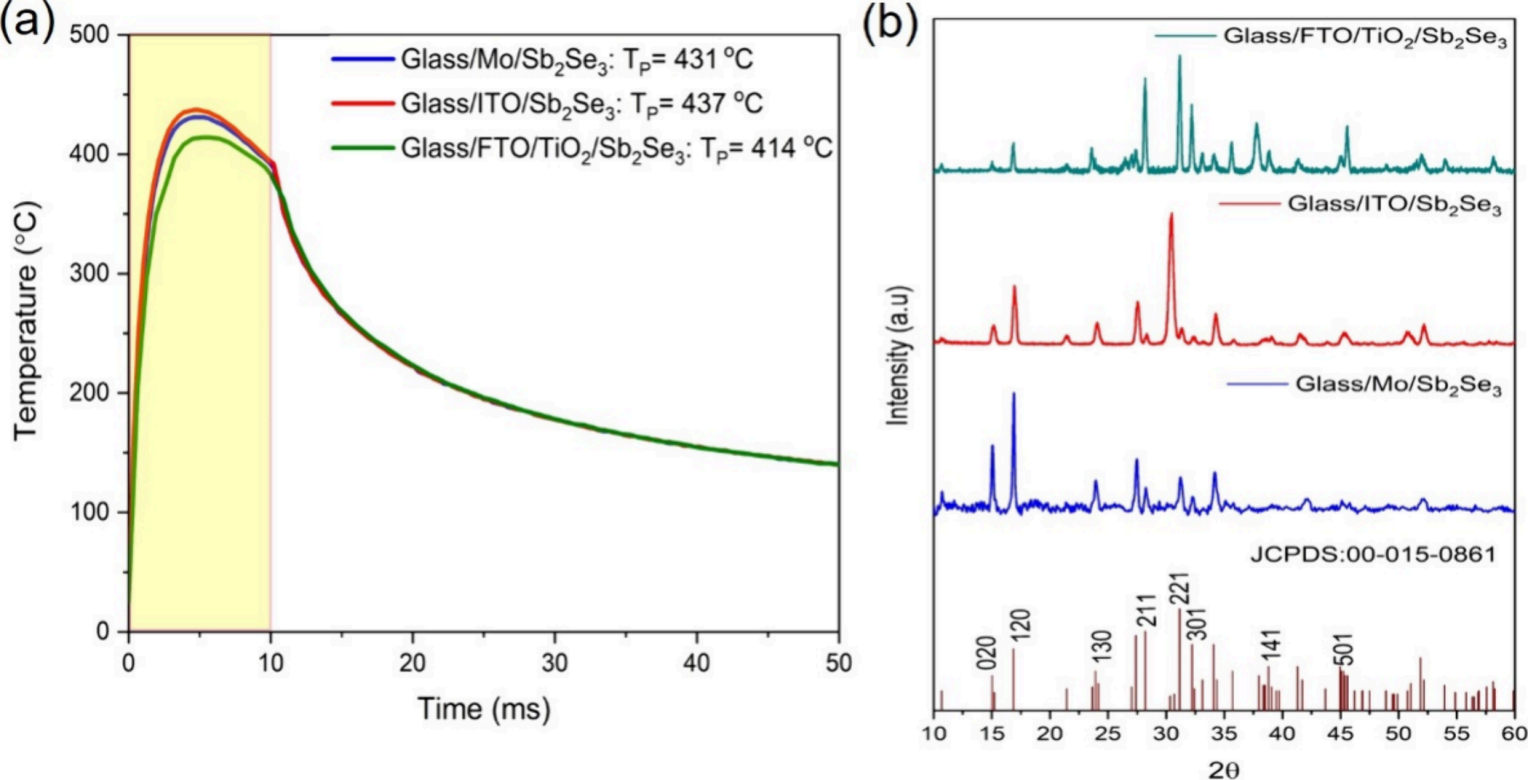
(a) Simulated temperature response of Sb_2_Se_3_ on glass/Mo, glass/ITO, and glass/FTO/TiO_2_ substrates after the onset of the 10 ms pulse (the yellow shaded region indicates the time the lamp is on). (b) XRD patterns of 1 μm PC Sb_2_Se_3_ deposited on glass/Mo (bottom, blue), glass/ITO (middle, red), and glass/FTO/TiO_2_ (top, green). PC conditions: 10 ms, 337 V, and 5 J cm^–2^.

Effectiveness of photonic curing depends on the pulse length, radiant energy, and heat transfer through the material. Heat transfer through the material and, therefore, optimum film temperature are determined by the optical properties, specific heat capacity, thermal conductivity, density, and thickness of all materials in the layer stack.^23,24^ To understand the effect of partner layers on the crystallinity of the absorber layer, Sb_2_Se_3_ films used in this work were deposited on three stacks: glass/Mo, glass/ITO, and glass/FTO/TiO_2_. Mo and TiO_2_ are the most used partner layers for substrate and superstrate photovoltaic devices, respectively.^6,7,25^Figure 2a shows the simulated temperature profiles for Sb_2_Se_3_ on Mo, ITO, and TiO_2_ substrates over a pulse length of 10 ms with 5 J cm^–2^ (fabrication procedures are given in the Supporting Information). Table S1 gives the thermal constants used for the temperature simulations. The thermal diffusivity of the Sb_2_Se_3_ thin film, calculated by the ratio of the thermal conductivity (κ = 0.22 W m^–1^ K^–1^)^26^ to the volumetric heat capacity (ρCp = 1528.2 J cm^–3^ K^–1^),^27^ is 1.44 × 10^–6^ cm^2^ s ^–1^. At pulse durations of 1 and 10 ms, the thermal diffusion lengths of the film are 0.76 and 2.4 μm, respectively, which are larger than the thickness of the deposited film for the 10 ms case. For the 10 ms pulse, this would suggest fast thermal diffusion through the film. Since the amount of energy delivered to the three samples is the same, the peak temperature (*T*_P_) of the Sb_2_Se_3_ film on each substrate depends on the heat transfer process. Figure 2a shows that as soon as the lamp turns on, the film temperature sharply increases, and *T*_P_ ∼ 431 °C (glass/Mo/Sb_2_Se_3_), *T*_P_ ∼ 437 °C (glass/ITO/Sb_2_Se_3_), and *T*_P_ ∼ 414 °C (glass/FTO/TiO_2_/Sb_2_Se_3_) are reached within a few milliseconds. After that, the Sb_2_Se_3_ layers rapidly cool to temperatures below 100 °C within 100 ms due to the heat transfer into the cold glass substrate (25 °C). The variation of crystallization of 1 μm Sb_2_Se_3_ on three substrates was analyzed via XRD (Figure 2b). The provided energy of 5 J cm^–2^ crystallized Sb_2_Se_3_ on TiO_2_ with mainly (211) and (221) orientations (as discussed below), while the Mo and ITO substrates promote (020) and (120) growth. The (211) and (221) planes indicate that the ribbons inclined at 37° and 44° relative to the substrate normal, respectively, which enhances the charge transport through the ribbons, whereas (020) and (120) planes consist of ribbons parallel to the substrate, giving poor conductivity as excited carriers must hop between neighboring ribbons.^6,25^ Although the simulated *T*_P_ values of Mo and ITO substrates are similar, Sb_2_Se_3_ on the ITO substrate was completely delaminated due to poor adhesion. Figure S1 demonstrates the color of the amorphous Sb_2_Se_3_ changes upon PC and shows variation in the adhesion of Sb_2_Se_3_ films on different substrates. The comparable *T*_P_ on different substrates leads to very different morphologies, as shown in Figure S2. This shows that the film stack plays a crucial role in determining the correct *T*_P_ and, therefore, the relevant PC conditions for a given material. For further analysis of PC conditions, Sb_2_Se_3_ films on the glass/FTO/TiO_2_ substrate were selected, and Figure 1d summarizes the experimental results of PC as a function of pulse length and measured radiant energy from the bolometer: yellow, green, and red regions represent PC conditions that produce underconverted (amorphous), converted (crystalline), and overconverted (delaminated) Sb_2_Se_3_ films, respectively. For the purposes of this study, we have defined the threshold between underconverted and converted as displaying any Sb_2_Se_3_ peaks in an XRD pattern and the threshold between converted and overconverted as when more than 1% of the film has been ablated from the surface.

It can be observed that at a fixed pulse length, more radiant energy delivered to the sample increases the conversion of Sb_2_Se_3_ films, while the conversion region enlarges as the pulse length increases. This can be explained with reference to the average radiant power. As observed in Figure 1c, the average radiant power of shorter pulses is more intense than that of longer pulses. Furthermore, a shorter discharge of the lamp delivers a higher current density in the UV range, which explains why overconverted films are easily obtained (i.e., the Xe lamp spectrum depends on the current density).^28^

**Figure 3:**
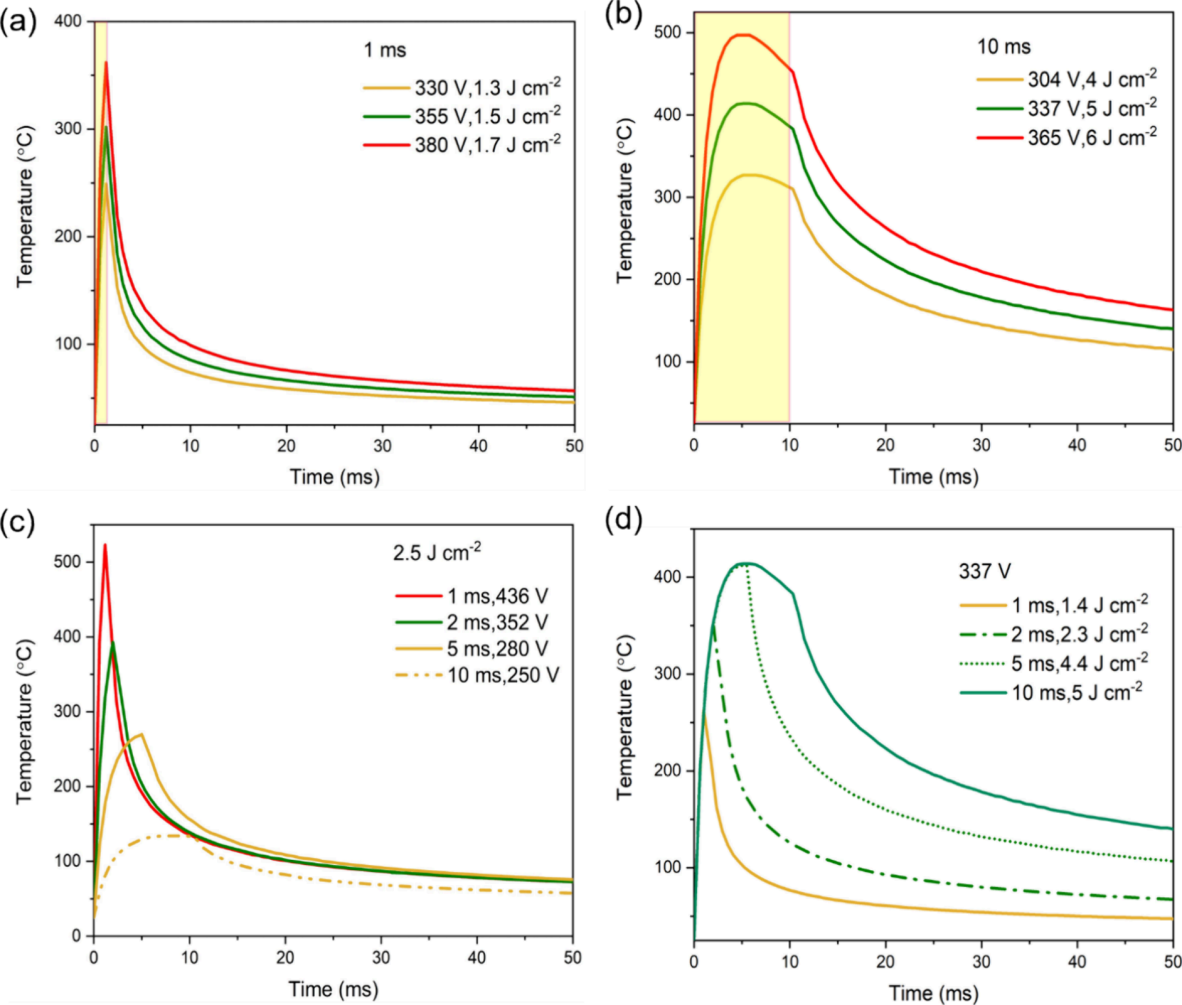
SimPulse-simulated temperatures at the Sb_2_Se_3_ surface versus time at fixed pulse lengths of (a) 1 and (b) 10 ms with different radiant energies. (c) Simulated temperature profiles at 2.5 J cm^–2^ radiant energy with different pulse lengths. (d) Simulated temperature profiles at 337 V lamp voltage with different pulse lengths. The outcome of Sb_2_Se_3_ films processed using a specific PC condition is shown in yellow, green, and red to represent underconverted, converted, and overconverted films, respectively.

To further understand these results, the temperature response of Sb_2_Se_3_ films was calculated from SimPulse for varying radiant energies at fixed pulse lengths of 1 and 10 ms (Figure 3a,b), for varying pulse lengths at a fixed radiant energy of 2.5 J cm^–2^ (Figure 3c), and for varying pulse lengths at a fixed lamp voltage of 337 V (Figure 3d). For all conditions, the peak temperature remains below the melting point of Sb_2_Se_3_ (611 °C). For a constant pulse length, an increase in the radiant exposure results in an increase in power, eventually resulting in damage to the film due to increased power delivery and higher surface temperatures of the sample. With a short pulse length (Figure 3a), the film temperature rapidly rises when the lamp is on and then decreases immediately when the lamp is off, resulting in a steep rise and fall in the film temperature. This creates the differential thermal mass distribution between the Sb_2_Se_3_ film and the substrate, which results in significant thermal damage to the Sb_2_Se_3_ film (thermal expansion coefficient of Sb_2_Se_3_ of 3.7 × 10^–5^ K^–1^ and that of TiO_2_ of 11.8 × 10^–6^ K^–1^).^29,30^ On the other hand, under a long pulse length (Figure 3b), the film temperature initially rises quickly and then decreases gradually while the light is still on. Then, the temperature was decreased to room temperature when the light is turned off. In the PC system used in this study, the flash lamp is powered by the capacitor bank, which is drained during long pulses, resulting in a decreased lamp power output. The gradual decrease in film temperature while the lamp is on is due to the film cooling rate exceeding the lamp power output. For example, for a 10 ms lamp onset, *T*_P_ occurs at ∼5 ms, at which point the heating rate from the absorption of lamp energy and the cooling rate due to heat loss to the substrate and the environment are balanced. Therefore, longer pulses cause less sample surface damage compared to shorter pulses.

In contrast, for fixed radiant energy, an increase in the pulse length decreases the temperature reached by the film (Figure 3c). The delivered lamp power is calculated as the integrated radiant energy over the pulse length. Therefore, for the same radiant energy (J cm^–2^), the average radiant power (W cm^–2^) received by the sample will be lower for longer pulses.^28^ This is clearly evidenced by the reduction in the maximum temperature when the pulse length is increased. Finally, Figure 3d shows the temperature variation at fixed lamp voltage. The overlap in the rise in temperature of the different pulse lengths is noticeable, with a higher temperature achieved by increasing the pulse duration. This is due to the fact that when the same voltage is used, the generated current (and, therefore, the pulse) profile is the same, only kept for longer times.^28^

To better understand the effects of pulse length and radiant energy on the crystallinity and morphology of Sb_2_Se_3_ films, three different regions of the “converted area” in Figure 1d were selected. Inside that region, low (L), medium (M), and high (H) values of radiant energy for 1, 5, and 10 ms pulse lengths were chosen. The corresponding PC conditions used in this study are summarized in Table 1.

**Table 1:** Detailed Processing Conditions of the Sb_2_Se_3_ Thin Films

sample	annealing method	pulse length (ms)	lamp voltage (V)	radiant energy (J cm^–2^)	radiant power (W cm^–2^)	peak film temperature (°C)
TA-V	TA	N/A	N/A	N/A	N/A	400
TA-N	TA	N/A	N/A	N/A	N/A	400
1 ms-L	PC	1	347	1.5	1.5	284
1 ms-M	PC	1	364	1.55	1.55	323
1 ms-H	PC	1	382	1.7	1.7	362
5 ms-L	PC	5	325	3.8	0.78	388
5 ms-M	PC	5	338	4.2	0.84	412
5 ms-H	PC	5	350	4.6	0.92	450
10 ms-L	PC	10	321	4.5	0.45	370
10 ms-M	PC	10	337	5.0	0.50	414
10 ms-H	PC	10	352	5.5	0.55	455

**Figure 4:**
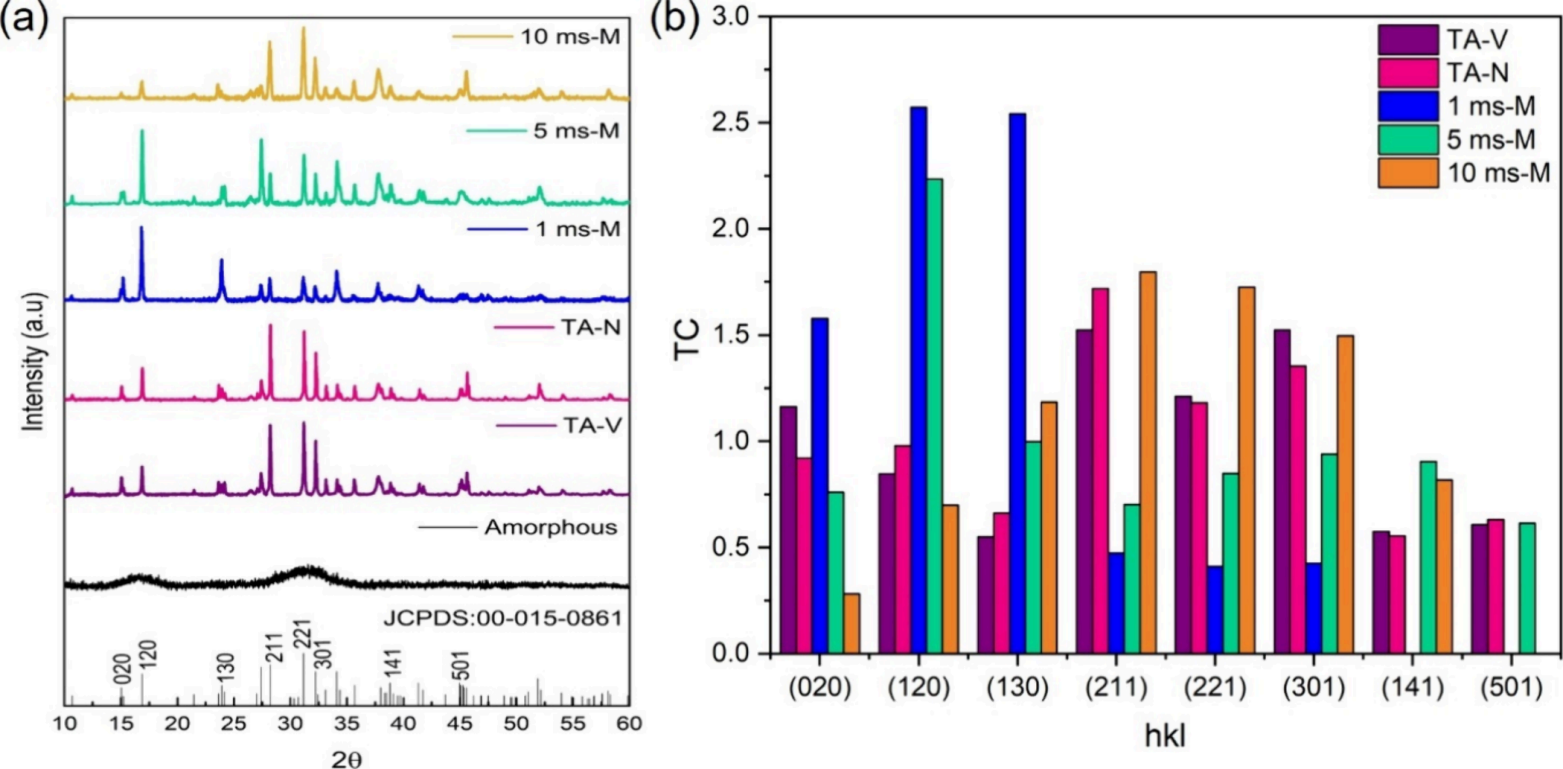
(a) XRD patterns of reference, amorphous, TA, and PC Sb_2_Se_3_ films. (b) Texture coefficients of the Sb_2_Se_3_ thin films processed under the conditions mentioned in Table 1.

Optimization of radiant energy based on crystallinity and growth orientation of the Sb_2_Se_3_ films was performed by XRD analysis. In Figure 4, the samples treated with different pulse lengths at medium radiant energy are presented, together with the thermally annealed samples. All of the diffraction patterns were indexed to orthorhombic Sb_2_Se_3_ (JCPDS no. 015-0861). The films annealed under TA-V and TA-N exhibit the desired strong (211) and (221) peaks with some differences in peak intensities. The films cured under 1 ms-M and 5 ms-M mainly promote (020) and (120) orientations, while 10 ms-M promotes the desired preferred orientation of (211) and (221). The (020) and (120) planes possess surface energies lower than those of the (211) and (221) planes.^25^ When PC is performed, induction of a high energy pulse of light results mainly in low energy growth along the (020) and (120) planes. Figure S3 shows that by changing the energy from low to high, the orientation of Sb_2_Se_3_ changes significantly, and higher energy, irrespective of pulse length (1 ms-H, 5 ms-H, and 10 ms-H), will result in significant film damage, inducing more pin holes (Figure S4). In this context, it must be remembered that the portion of UV emission of a discharge Xe lamp increases with increasing capacitance voltage,^28^ which may result in additional damage to the thin films. The grain orientations were distinct when the pulse length increased from 5 to 10 ms-M, as can be observed in Figure 4a, as more time is allowed for the atoms’ reorganization, promoting grains with high crystallinity and vertical orientation. It is worth noting that no peaks pertaining to an impurity phase, such as Sb_2_O_3_, elemental Sb, or elemental Se, were observed within the detection limit of XRD.

To quantify the difference in crystal orientations, the texture coefficient (TC) of diffraction patterns of these samples annealed under different conditions was calculated using the equation^31^:
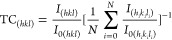
where *I*_(*hkl*)_ and *I*_0(*hkl*)_ are the diffraction peak intensities of the (*hkl*) planes in the measured and standard XRD patterns of Sb_2_Se_3_ (JCPDS no. 00-015-0861), respectively.

A high TC value for a diffraction peak indicates a preferred orientation of the grain along a particular crystal plane compared to ideal values. As shown in Figure 4b, the TC values of the (211) and (221) planes are high, whereas the TC values of the (020) and (120) planes are low for TA samples. When the pulse length increases from 1 to 10 ms, the TCs of (211) and (221) increased with a concomitant decrease in (020) and (120) planes. The difference between these results demonstrates that the pulse length and radiant energy were crucial in orientation preference. Due to the quasi-one-dimensional crystal structure of Sb_2_Se_3_, films with the (*hkl*, *l* ≠ 0) orientations (i.e., oriented in the (211) and (221) directions) are preferred for PV applications due to better carrier transport and collection through the absorber layer.^4−6^ Therefore, tuning PC parameters for a 10 ms pulse length can form films with these preferred orientations.

**Figure 5:**
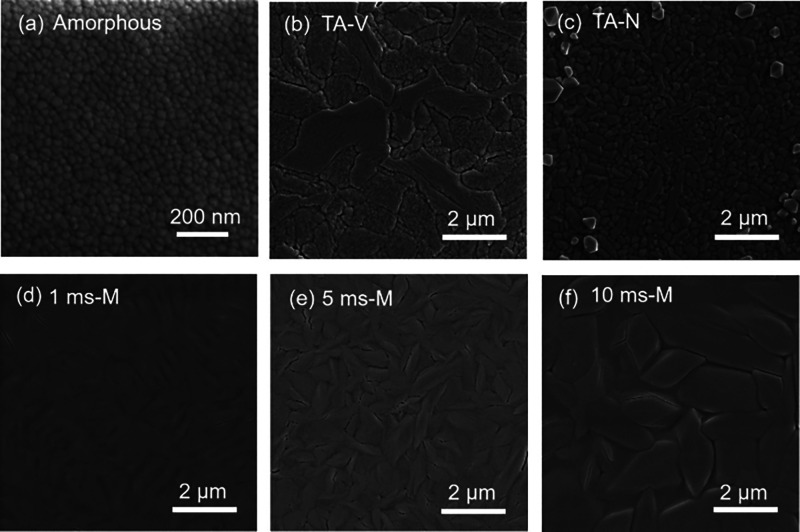
SEM images of Sb_2_Se_3_ films prepared under postdeposition annealing conditions indicated in Table 1. (a) Amorphous, (b) TA-V, (c) TA-N, and (d) 1, (e) 5, and (f) 10 ms-M.

SEM was performed to understand the effect of PC conditions on the Sb_2_Se_3_ film morphology. Figure 5 shows top-view SEM images of amorphous, TA-V, TA-N, 1, 5, and 10 ms-M films. The SEM micrograph for the as-deposited film shows a smooth surface with structures on the order of tens of nanometers with an amorphous nature, as evidenced by the XRD pattern of these films (Figure 4a). TA samples present densely packed grains, confirming the good crystallinity of the films, which is consistent with the XRD results. TA-V shows dominant grain growth and exceptionally large grains compared with the other films (Figure 5b). TA-N samples show additional structures on top of Sb_2_Se_3_, which are discussed in a later section. The change in surface morphology of Sb_2_Se_3_ when increasing the pulse length is shown in Figure S5. The crystallinity and average grain size of Sb_2_Se_3_ increase with an increase in the radiant energy, suggesting reduced nucleation and higher growth rates. The PC films show more continuous grain growth compared to TA, which is composed of numerous small clusters. It can also be noted how this distinctive structure is promoted as the PC pulse length is increased, with 5 ms-M producing larger and more compact grains than 1 ms-M and with 10 ms-M presenting a similar grain shape with the biggest size over 2 μm. When moving from the mid- to high energy, irrespective of pulse length (e.g., 1 ms-M to 1 ms-H), damage to the surface, such as pinholes, is observable. It is worth mentioning that during slow TA, grains have more time to grow into large, oriented crystals; however, by comparison, photonically annealed films are crystallized in timeframes of 2.7 million times faster (45 min vs 10 ms) using entirely different crystallization kinetics, thus leading to larger grain sizes. The measured thickness of 1 μm for as-deposited films is retained after annealing treatments, showing no ablation of the film thickness during postannealing treatments (Figure S6).

The SEM images of TA-V (Figure 5b) and 10 ms-H (Figure S4i) show the microstructure within the grain interiors (step bunching). To our knowledge, no detailed study on these microstructures in Sb_2_Se_3_ films has been performed, but similar features have been reported on perovskite films processed with PC.^13^ Step bunching occurs due to the misorientation between the grains’ fastest growth direction and the surface normal. Among the PC samples, step bunching is promoted under higher radiant energies. However, the TA-V sample exhibits more prominent step bunching compared with that of 10 ms-H.

The compositional analysis obtained via EDS for each film is summarized in Table 2. Amorphous Sb_2_Se_3_ thin films are slightly Se-rich with a Se/Sb ratio of 1.60 (for stoichiometric Sb_2_Se_3_, Se/Sb = 1.50). This may be due to the deposition of a more volatile Se-rich final layer when the thermal source cools on a thermal evaporation run of Sb_2_Se_3_. The PC thin film shows the same composition as the amorphous films within error, confirming that during PC, there is no Se loss and the composition of Sb_2_Se_3_ is maintained. Therefore, the use of PC can avoid formation of Se-deficient Sb_2_Se_3_ films and, thus, formation of Se vacancies, which would act as deep recombination centers,^1^ potentially avoiding an additional postselenization step in Sb_2_Se_3_ film fabrication. The TA films revealed a loss of the more volatile Se due to annealing at higher temperatures for a long time. It is important to note that EDS point measurements of TA-N show a very low-intensity oxygen peak. EDS mapping of the TA-N sample (Figure 5c) confirms the presence of Sb_2_O_3_ on top of the sample (Figure S7). This observation demonstrates that the detected oxygen signal originates from large crystals of Sb_2_O_3_. All the single point and mapping EDS analysis was performed in high resolution mode, at low energy and with a calibrated electron beam, to improve the accuracy of the quantitative results and the map lateral resolution. Furthermore, Raman spectra show the known major modes of Sb_2_Se_3_ for all samples and Sb_2_O_3_ for only the TA-N sample (Figure S8). The oxide formed on the surface was found to be α-Sb_2_O_3_, which is the most common Sb oxide formed.^32^ Both α-Sb_2_O_3_ and α-monoclinic Se (S8 ring structure) display a major Raman peak at 255 cm^–1^.^32,33^ No secondary phases were found in any of the other films. During measurements, the laser power and exposure times to the samples were minimized to prevent sample degradation.

**Table 2:** Statistical Composition Information by EDS Spectra

	composition (at %)		
sample	Se	Sb	Se/Sb
amorphous	61.6	38.4	1.60
TA-V	59.5	40.5	1.47
TA-N	54.6	45.5	1.20
1 ms-M	61.8	38.2	1.62
5 ms-M	61.6	38.4	1.60
10 ms-M	61.7	38.3	1.61

XPS analysis of amorphous and PC samples is presented in Figures S9 and S10. It must be noted that XPS has been performed on a thinner sample (300 nm) with different pulse treatments (3 pulses of 0.5 ms each, corresponding to 1.4 J cm^–2^ per pulse). The choice of a thinner sample was made to observe any possible interfacial diffusion. To ensure the heating of the entire film while keeping short pulses, 3 repetitions were performed. As shown in Figure S11, the simulated *T*_P_ is similar to the range of the one used with single pulses (10 ms-M), and the obtained film is crystalline without secondary phases, indicating the achieved crystallization of the amorphous film. Interestingly, the angle-dependent XPS measurement indicates the formation of surface oxide and elemental selenium only in the top few nanometers of the cured samples, which explains why EDS and Raman analysis did not detect these species. This has been previously shown for other Sb_2_Se_3_ surfaces,^34^ even if the extent of oxidation and, more specifically, selenium segregation in films subjected to PC is more significant than that of close space sublimation-deposited material.^32^ Band alignment improvements by these species, such as those described previously, may also improve the interfaces here, although care must be taken to avoid overoxidation, which has been shown to be harmful to device performance.^32^

AFM analysis was performed on these samples to quantitatively analyze topographical features. Figure S12 shows surface topographical images obtained from amorphous, TA-V, TA-N, and 1, 5, and 10 ms-M films. Root mean square (RMS) values of surface roughness are listed in Table 3, which demonstrates a linear relationship of RMS roughness with the pulse length, in which the 1 ms-M film represents a smooth surface with very minute grains with the lowest roughness of ∼2 nm similar to the amorphous sample, whereas 10 ms-M exhibits the largest grains and highest roughness of ∼14 nm, which is comparable with the TA-V sample. Individual grains visibly increase in size when pulse length/radiant energy increases. This may be due to the increased atom mobility, which leads to higher surface diffusion rates and agglomeration of smaller grains. However, at the same time, the increased surface mobility can also result in a higher propensity for surface roughness due to the formation of surface defects and non-uniformities during film growth. The larger TA-N roughness compared to those of the other samples can be ascribed to the formed surface α-Sb_2_O_3_.

**Table 3:** Surface Roughness of Sb_2_Se_3_ Samples Obtained from AFM Images

sample	surface roughness (nm)
amorphous	2.6 ± 0.1
TA-V	14.6 ± 2.1
TA-N	37.2 ± 0.9
1 ms-M	2.7 ± 0.2
5 ms-M	6.4 ± 0.2
10 ms-M	14.6 ± 3.1

**Figure 6:**
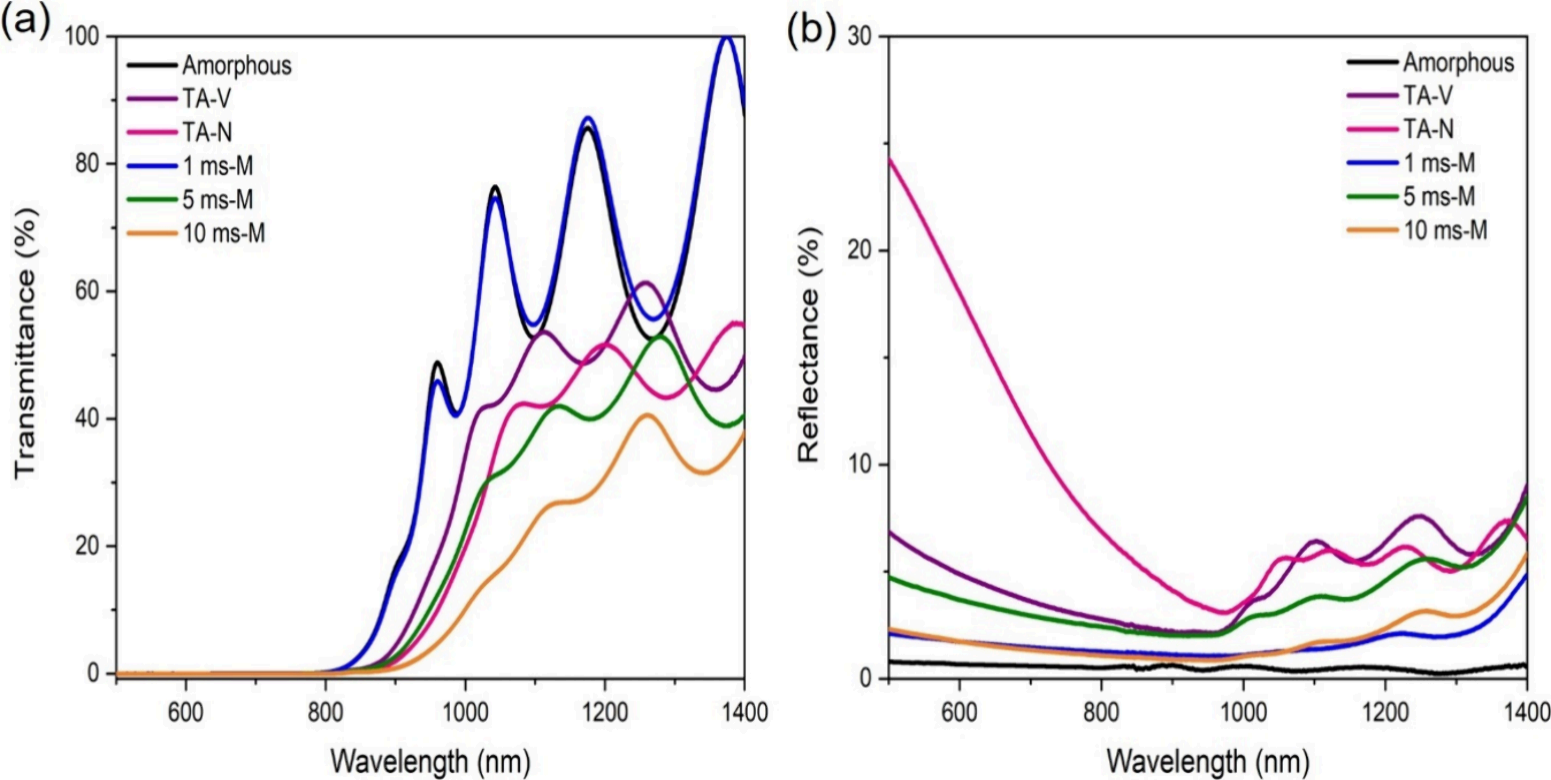
(a) Optical transmission spectra. (b) Optical reflection spectra of as-deposited and postdeposition-annealed Sb_2_S_3_ thin films.

Figure 6a shows the optical transmittance spectra measured in the 300–1400 nm wavelength range for as-deposited and Sb_2_Se_3_ films annealed under the conditions shown in Table 1. The optical transmittance of Sb_2_Se_3_ is higher in the near-infrared region than in the visible region, and all of the samples show a similar trend in transmittance. The transmittance spectra show interference fringes with a sharp fall at the band edge in the medium and weak absorption regions. It is interesting to observe that the PC films present a reflectance lower than that of TA, especially in the visible region (Figure 6b). The films showed good optical absorbance in the visible region, and this was observed to be increased for PC films. The plot for the absorption coefficient versus energy is shown in Figure S13b. The optical absorbance is highly sensitive to grain size variation and crystallinity improvement. When amorphous Sb_2_Se_3_ crystallizes, the absorption increases, and the absorption edge redshifts toward longer wavelengths, indicating that the band gap becomes narrower with postannealing treatment. An increase in the crystallinity with the consequent absorption redshift is shown for PC Sb_2_Se_3_ at a higher pulse length compared to a lower pulse length (Figure S13). Interestingly, absorption coefficients of 1.79 × 10^5^ and 1.62 × 10^5^ cm^–1^ (at 550 nm) were obtained for 5 ms-M and 10 ms-M, respectively, which are higher than TA samples (Table S2).

The optical band gap (*E*_g_) of Sb_2_Se_3_ thin films was determined using the empirical relation (α*h*ν)^*n*^ = *A*(*h*υ – *E*_g_).^35^ The approximately linear nature of the plot (*n* = 2) indicates the presence of direct transition in Sb_2_Se_3_ thin films, as expected. The optical energy band gap is found to be 1.63 eV for as-deposited amorphous films (Figure S14a), which is in good agreement with earlier reported work.^36−38^ The TA-V sample shows a band gap of 1.18 eV, which is similar to crystalline Sb_2_Se_3_.^37−39^ Meanwhile, a slightly higher band gap of 1.25 eV was obtained for TA-N. The reduction in the band gaps of the films after TA treatments is ascribed to the improvement in crystallinity, which is evidenced in the XRD patterns given in Figure 4. The band gap values for PC samples decrease from 1.40 to 1.27 eV upon an increase in the pulse length from 1 to 10 ms-M (Figure S14). The decrease in the band gap of cured films is attributed to the increased crystallinity. Band gap values of the PC films are expected to be slightly higher than the TA films as the PC films are more selenium-rich. However, the different band gap values across the PC samples, even if with a similar Se/Sb ratio, possibly indicate that incomplete crystallization is achieved with shorter pulses. This is also supported by the calculated heat diffusion length for the 1 ms pulse, which is smaller than the absorber thickness. The Urbach energy was calculated by fitting the exponential band-edge portion of the natural logarithm of the absorption coefficient versus the energy plot (Table S2), and it is within the range of previously reported values for this material.^39^ TA-V and 10 ms-M show similar magnitudes of localized states. This unusually large value may result from some regions in the Sb_2_Se_3_ film of lower crystallinity or minor variations in Se/Sb ratio affecting the band gap. The Urbach energies are not caused by secondary phases, as this has been ruled out with XRD patterns (Figure 4) and Raman spectra (Figure S8). Further optimization of growth conditions can therefore reduce the Urbach energies of the Sb_2_Se_3_ films.

**Figure 7:**
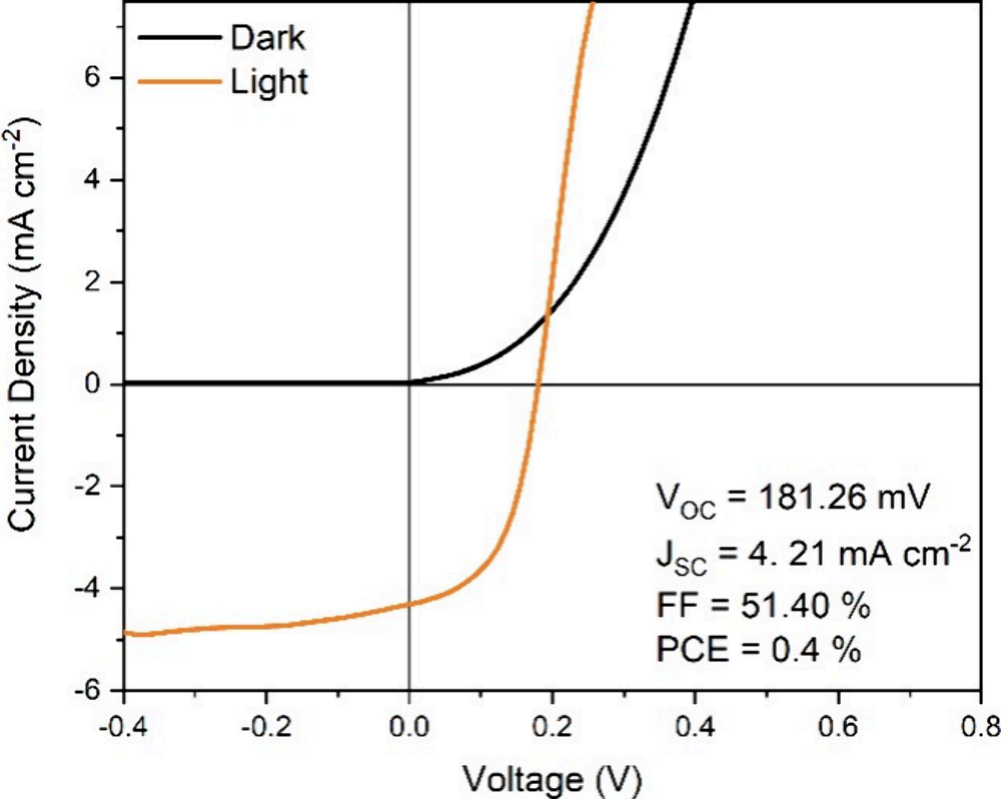
*J*–*V* curve of a solar cell fabricated with 10 ms-M PC Sb_2_Se_3_.

Finally, we present here the current–voltage characteristic of a Sb_2_Se_3_ solar cell with the absorber photonically cured with the most promising conditions identified in this work, that is, 10 ms-M (Figure 7). The PC device presents the characteristic rectifying behavior in the dark while showing voltage and current generation when characterized under illumination. It is important to stress that no optimization was performed on the devices, which is beyond the scope of this work. However, an initial open circuit voltage (*V*_OC_) of 181 mV and a fill factor of over 51% are promising. Additionally, the lack of S-kinks in the *J*–*V* curve indicates that PC is compatible with the solar cell fabrication process. Figure S15 indicates the *J*–*V* characteristics for both TA-V and TA-N Sb_2_Se_3_ solar cells. Given the very low performances obtained with the TA samples, Figure S15 also contains the *J*–*V* curve of an RTE device (prepared with the same Sb_2_Se_3_, TiO_2_, and P3HT precursors and with similar absorber thickness) as a more meaningful control. Comparing the TA and PC devices, it can be noted that the short circuit current (*J*_SC_) is the most limiting factor in the PC performances, being 33, 25, and 13% of TA-V, TA-N, and RTE *J*_SC_, respectively. On the other hand, the *V*_OC_ of the PC device corresponds to 83, 93, and 54% of the corresponding TA-V, TA-N, and RTE *V*_OC_. This suggests that the limiting factors derive from poor carrier extraction rather than from increased nonradiative recombination or poor photogeneration (Figure S13, absorption coefficients above 10^5^ cm^–1^). This could be ascribed to noncomplete crystallization down to the buried interface that could reduce band alignment with TiO_2_. Similarly, interface defects could be an additional limitation to both *J*_SC_ and *V*_OC_ in PC solar cells, besides the possible presence of pinholes, which increases the number of shunting pathways. Device performance could be improved in the future by investigating multiple pulses, incoming light direction, and the use of different transport layers.

## 4 Conclusions

For the first time, this work presents the influence of PC parameters for crystallization of Sb_2_Se_3_ thin films deposited by thermal evaporation for optoelectronic applications. The film quality and topology of Sb_2_Se_3_ were found to be highly dependent on the PC conditions. The conversion range of Sb_2_Se_3_ was found to be narrow and strongly dependent on the pulse length and radiant energy. Pulse lengths >5 ms were attributed to more gentle temperature profiles and produced films with denser morphology and less surface damage. Moreover, long pulse lengths significantly influenced the preferred growth along the (211) and (221) planes with a TiO_2_ underlayer. Crucially, these are the near vertical orientations that are required for efficient charge transfer in optoelectronic devices and are combined with large grain size, ability to maintain stoichiometry due to the rapid heating process, and little film damage. The good optical properties of the photonically cured films, combined with the simple, low-cost, and easily scalable annealing technique to produce crystalline thin films, should prove very useful for optoelectronic applications, including solar cells. This is demonstrated with a proof-of-concept photovoltaic device. This new method achieves crystallization of antimony chalcogenide films millions of times faster than thermally annealed films with greater energy efficiency and, therefore, shows great potential and warrants further study.

## Supporting Information Available

Radiant power approximation, Mo and ITO substrate fabrication, and RTE device fabrication (PDF)
