# Correction to “Crystalline Antimony Selenide Thin Films for Optoelectronics through Photonic Curing”

**DOI:** 10.1021/acs.chemmater.4c02495

**Published:** 2024-11-29

**Authors:** Udari Wijesinghe, William D. Tetlow, Pietro Maiello, Nicole Fleck, Graeme O’Dowd, Neil S. Beattie, Giulia Longo, Oliver S. Hutter

**Affiliations:** † Department of Mathematics, Physics, and Electrical Engineering, Northumbria University, Newcastle upon Tyne NE1 8QH, United Kingdom; ‡ Jaguar Landrover, Banbury Road, Gaydon CV35 0RR, United Kingdom

In the original article ofChem. Mater.2024, 36, *12*, 6027–603738947981 10.1021/acs.chemmater.4c00540PMC11209937, an oversight occurred whereby an important reference (reference 13 in the original text) was cited but not properly discussed. The authors offer their apologies for this oversight. The reference provides important and valuable methodological information using photonic curing, and it was therefore taken as inspiration. In fact, the articleChem. Mater.2024, 36, *12*, 6027–603738947981 10.1021/acs.chemmater.4c00540PMC11209937 follows a similar methodology to the one reported in reference 13, even if applied to a different material system.

On page 6027, after the sentence citing reference 13, the above reference should be cited again with an additional sentence reading “The current work follows a methodology similar to the work from Xu et al.^13^”

On page 6031 after the sentence “Furthermore, a shorter discharge of the lamp delivers a higher current density in the UV range, which explains why overconverted films are easily obtained (i.e., the Xe lamp spectrum depends on the current density)”, the following sentence should be added: “Longer pulse lengths also work better on perovskites.^13^ which suggests that long pulse lengths could be beneficial for other light-absorbing photovoltaic materials.”

**Impact of the Addition**: This addition does not affect the conclusions of the original article in any way.

