# Supplementary material for: Crystalline Antimony Selenide Thin Films for Optoelectronics through Photonic Curing
Source: Chem Mater. 2024 Jun 7;36(12):6027–37. doi: 10.1021/acs.chemmater.4c00540 (PMC11209937; doi:10.1021/acs.chemmater.4c00540)
Supplement: Supplementary file 1 — cm4c00540_si_001.pdf [file cm4c00540_si_001.pdf]

Supplementary Information

Crystalline Antimony Selenide Thin Films for Optoelectronics through Photonic Curing

Udari Wijesinghe[1], William D. Tetlow[1], Pietro Maiello[1], Nicole Fleck[1], Graeme O'Dowd[2], Neil S. Beattie[1], Giulia Longo[1*], and Oliver S. Hutter[1*]

[1]Department of Mathematics, Physics, and Electrical Engineering, Northumbria University, Newcastle upon Tyne NE1 8QH, United Kingdom

[2]Jaguar Landrover, Banbury Road, Gaydon, CV35 0RR, United Kingdom

*To whom correspondence should be addressed: g.longo@northumbria.ac.uk and oliver.hutter@northumbria.ac.uk

Table S1. Simulation parameters used in SimPulse®.

Material	Thermal conductivity (W m^{-1} K^{-1})	Density (g cm^{-3})	Specific heat (J kg^{-1} K^{-1})	Melting point (°C)	Heat of fusion (kJ/mol)	Boil temperature (°C)	Heat of vapor (KJ/mol)	Molecular weight (g/mol)	Attenuation (cm^{-1})
Sb_2Se_3	0.22[1]	5.81[2]	262.69[3]	611[2]	1♠	1031.90#	60♠	480.40#	550000♠
TiO_2*	8.40	4.23	325.54	1843	293	2972	800	79.87	10
FTO*	12	6.85	1000	1630	106	1900	1	150.70	2
Soda lime glass*	1.10	2.52	800	1040	0.50	2400	1	226	0.33
ITO*	11	6.80	357	1913	105	982	1	277.60	2
Mo*	138	10.28	250	2623	37.48	4639	617	95.96	1000

(*Based on SimPulse® database, ♠Calculated, and #Chemical supplier label)

Radiant Power Approximation

The decay of lamp power over time results from the instrument's capacitor bank draining as the flash bulb is engaged. It was found that "rectangular" energy profiles could only be achieved using short pulse lengths and lower voltages than those required to anneal Sb_2Se_3 films in a single pulse duration fully. Such uniform profiles would reduce the complexity of obtaining optimal flash annealing parameters. However, when determining radiant power here, we assume the radiant energy is uniform throughout the pulse, and the pulse is rectangular in all the pulse lengths.

Mo and ITO Substrate Fabrication

Soda lime glass and ITO-coated glass substrates (Sigma Aldrich) were cleaned using sequential ultrasonic baths of DI water, acetone, and IPA, followed by UV-Ozone (Ossila) treatment for 15 minutes. Mo was sputtered onto glass using a Minilab 125 (Moorfield, UK) to prepare a bilayer film by a two-step DC magnetron sputtering process, which consisted of high working pressure (0.4 Pa) and low working pressure (0.13 Pa) steps at 200 ℃. The total thickness of Mo was 1 µm. Sb_2Se_3 absorber films were deposited on Mo and ITO coated glass substrates by thermal evaporation (Univex 250 special, Oerlikon Leybold) at a pressure of 1×10^{-6} mbar to obtain 1 µm Sb_2Se_3 films.

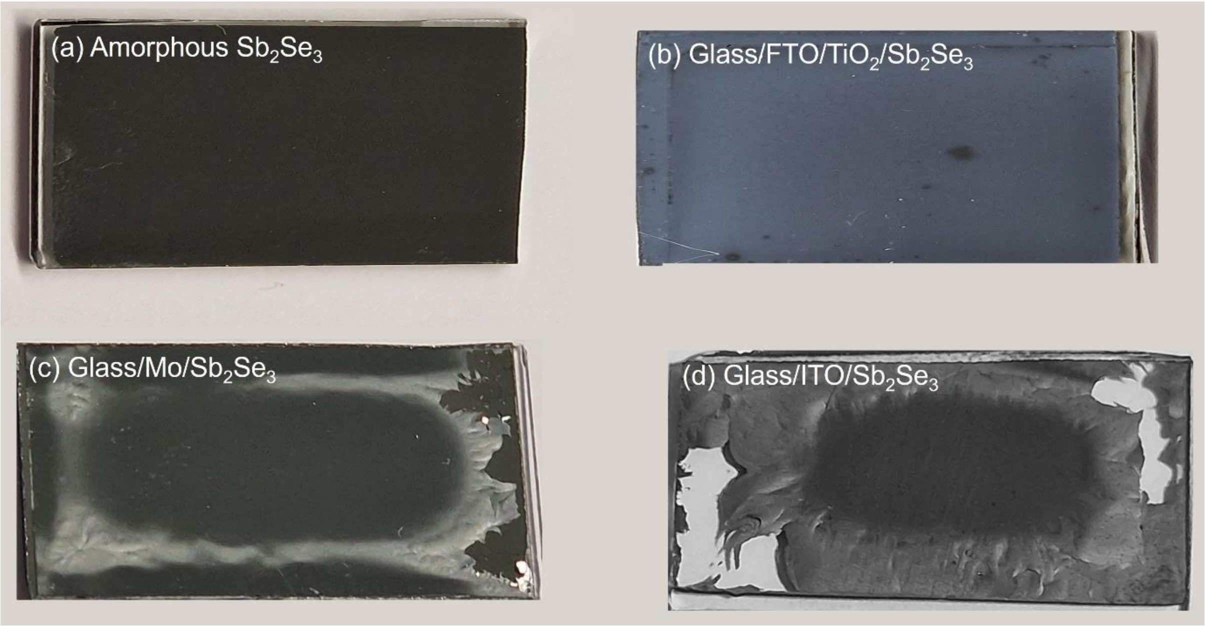

Figure S1. (a) Photograph of as-deposited Sb_2Se_3 on Glass/FTO/TiO_2 substrate. Photographs of PC processed Sb_2Se_3 on (b) Glass/FTO/TiO_2 substrate, (c) Glass/Mo, and (d) Glass/ITO. Sb_2Se_3 films undergo a change in colour from black in the as-deposited state to dark gray after PC.

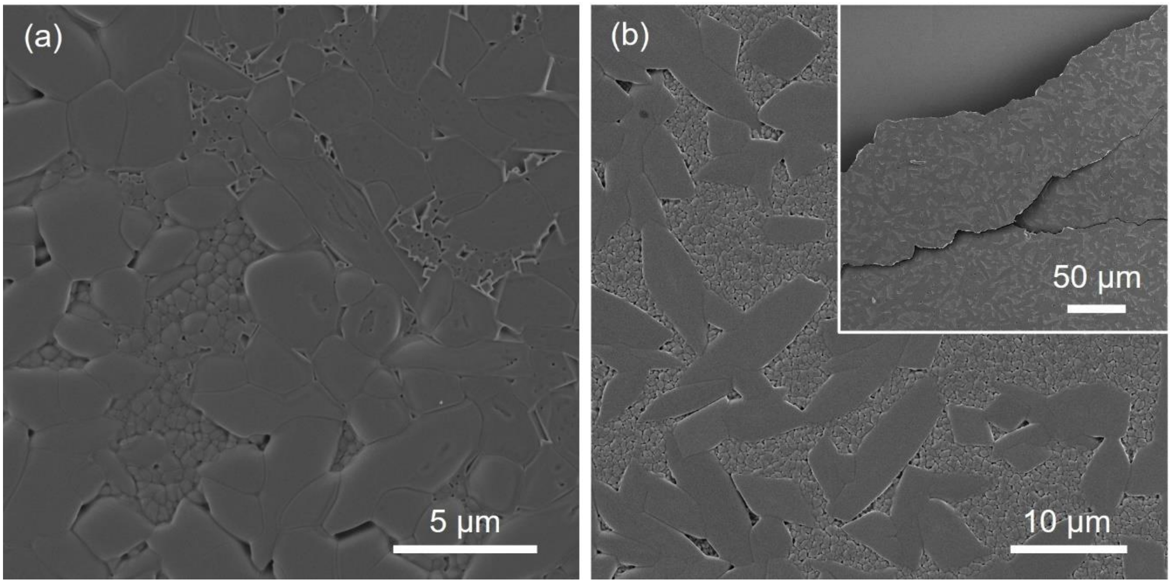

Figure S2. Top-view SEM images of PC Sb_2Se_3 on (a) Glass/Mo and (b) Glass/ITO (10 ms, 337 V, and 5 J cm^{-2}).

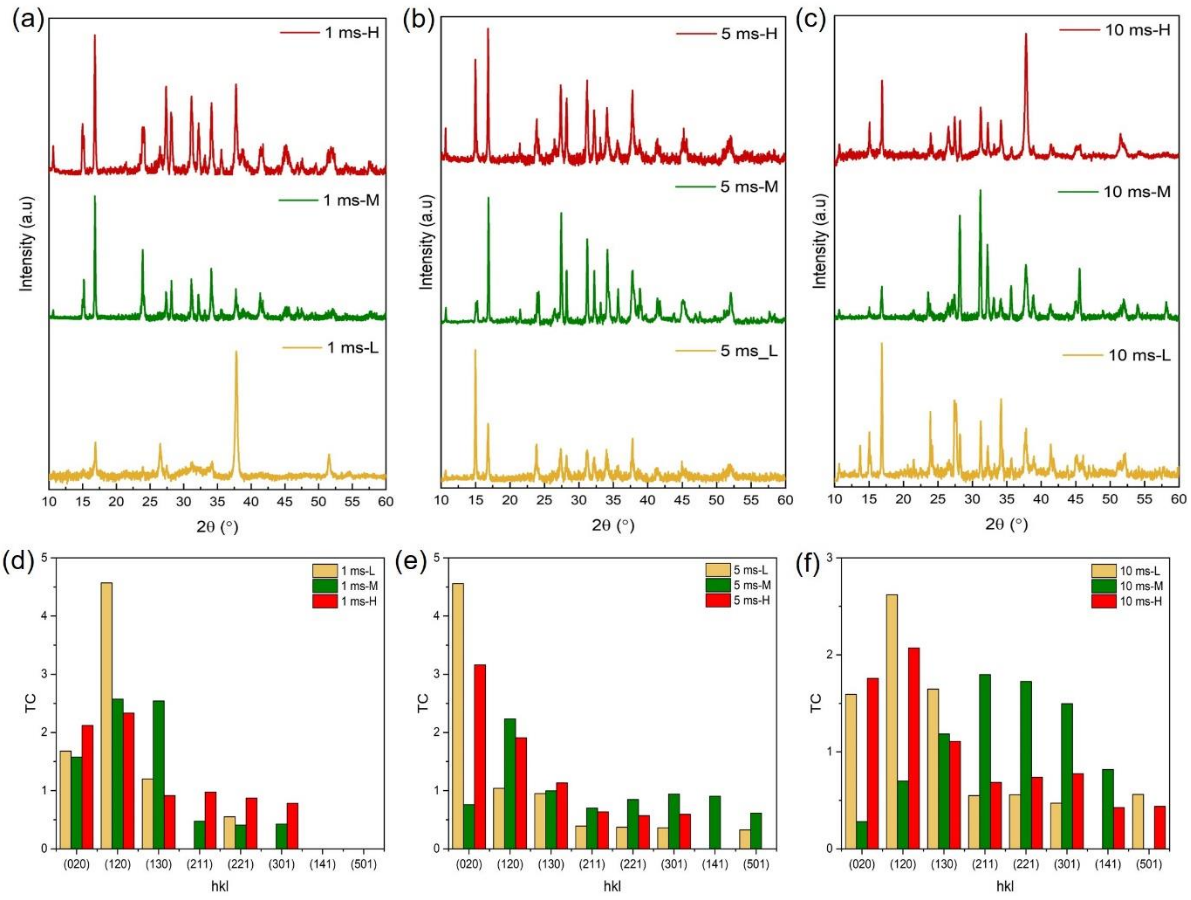

Figure S3. XRD patterns and texture coefficients of Sb_2Se_3 cured at fixed pulse lengths of (a,d) 1 ms, (b,e) 5 ms, and (c,f) 10 ms with different energy densities indicated in Table 1.

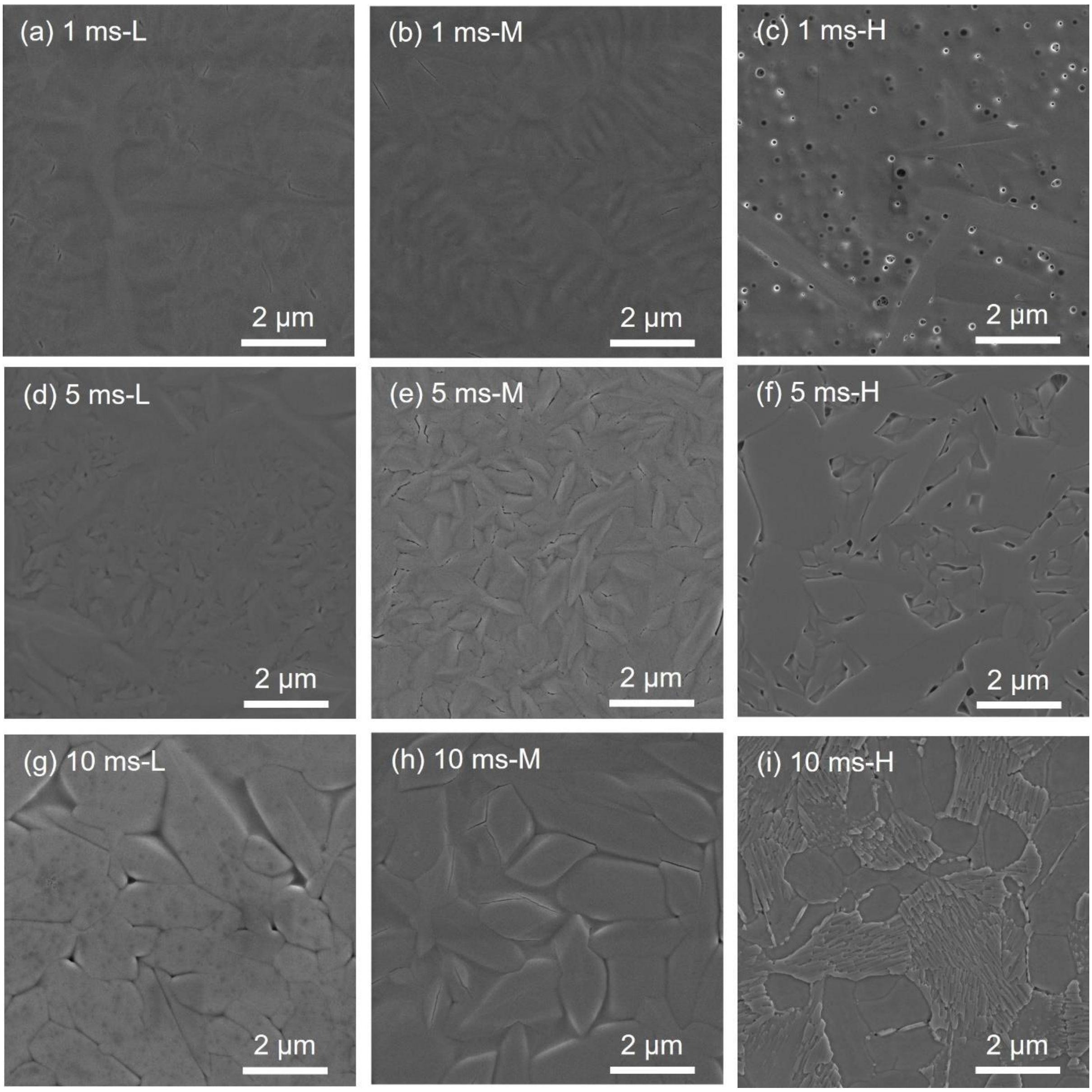

Figure S4. SEM images of Glass/FTO/TiO_2/Sb_2Se_3 prepared by different PC processing conditions as indicated (Table 1).

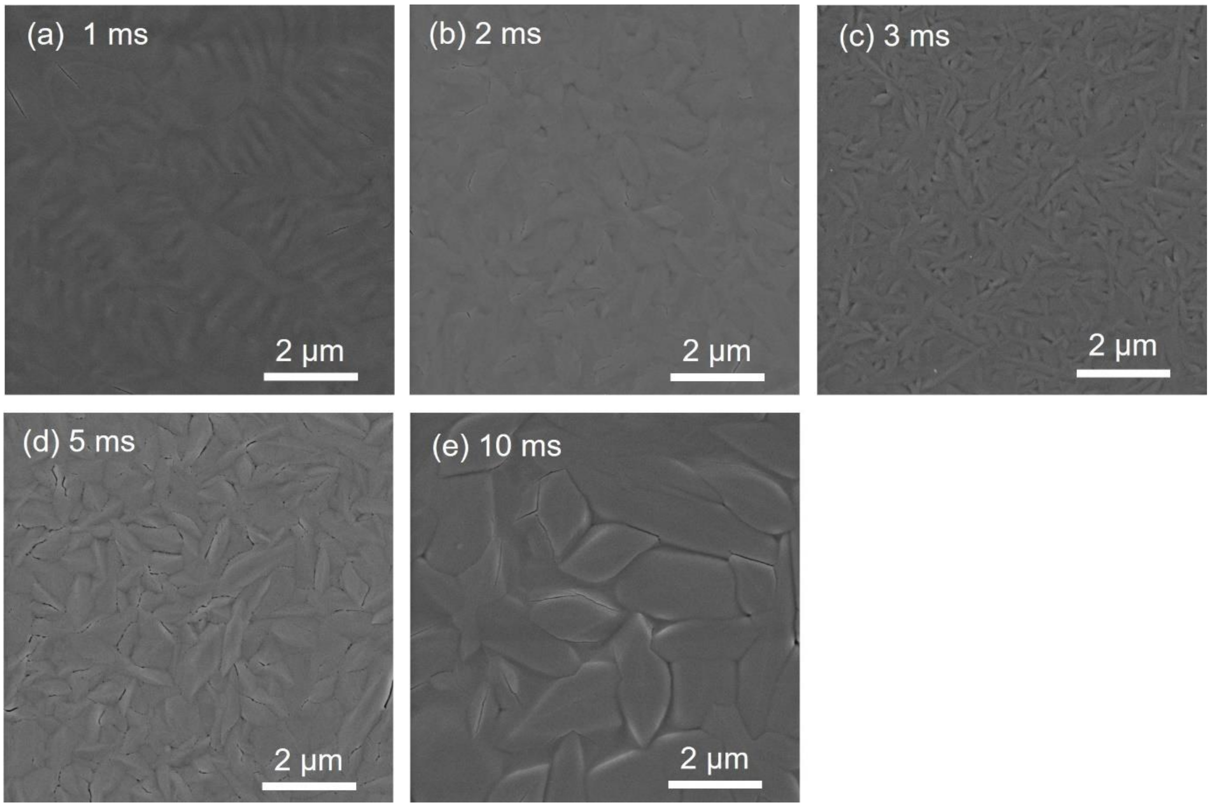

Figure S5. SEM images of Glass/FTO/TiO_2/Sb_2Se_3 films prepared by different pulse lengths (a) 1 ms, (b) 2 ms, (c) 3 ms, (d) 5 ms, and (e) 10 ms.

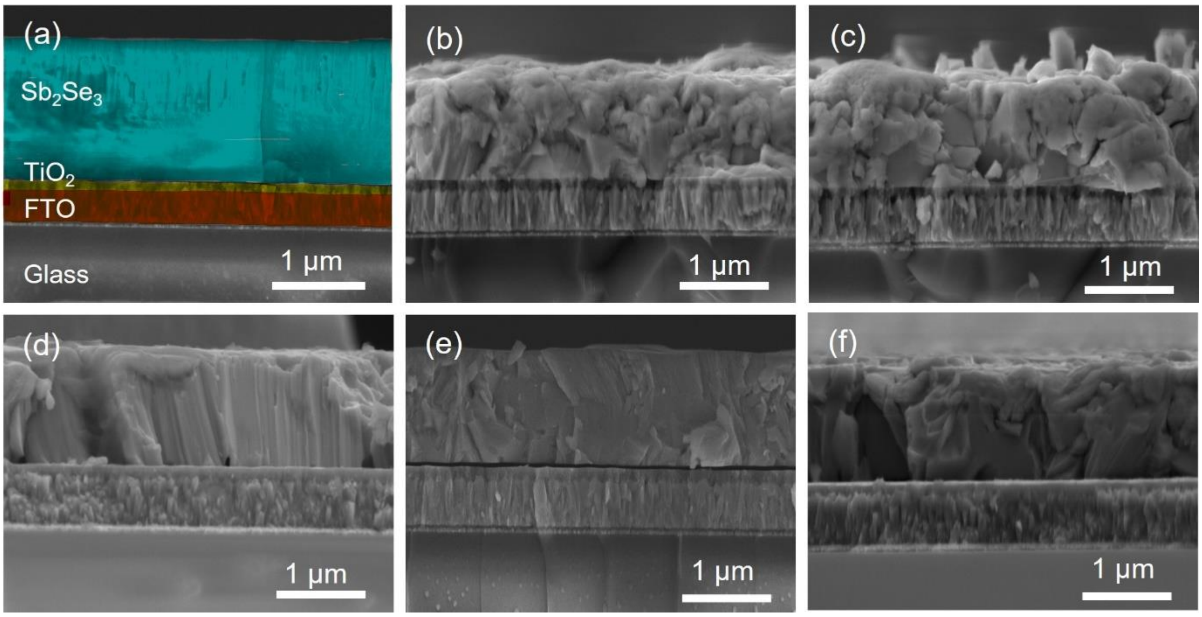

Figure S6. Cross-section SEM images of Glass/FTO/TiO_2/Sb_2Se_3 films (a) Amorphous, (b) TA-V, (c) TA-N, (d) 1 ms-M, (e) 5 ms-M, and (f)10 ms-M as indicated (Table 1).

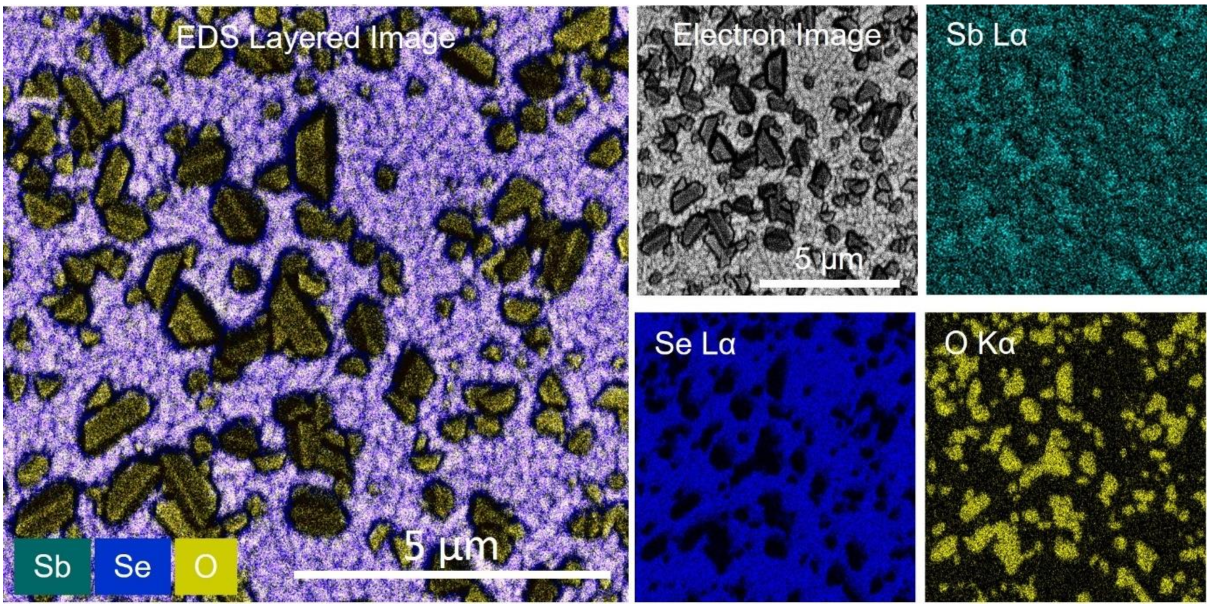

Figure S7. EDS mapping images of TA-N sample.

The Raman peak at 190 cm^{-1} is assigned to Sb−Se−Sb bending vibrations of Sb_2Se_3, and 255 cm^{-1} is attributed to α-Sb_2O_3.[4]

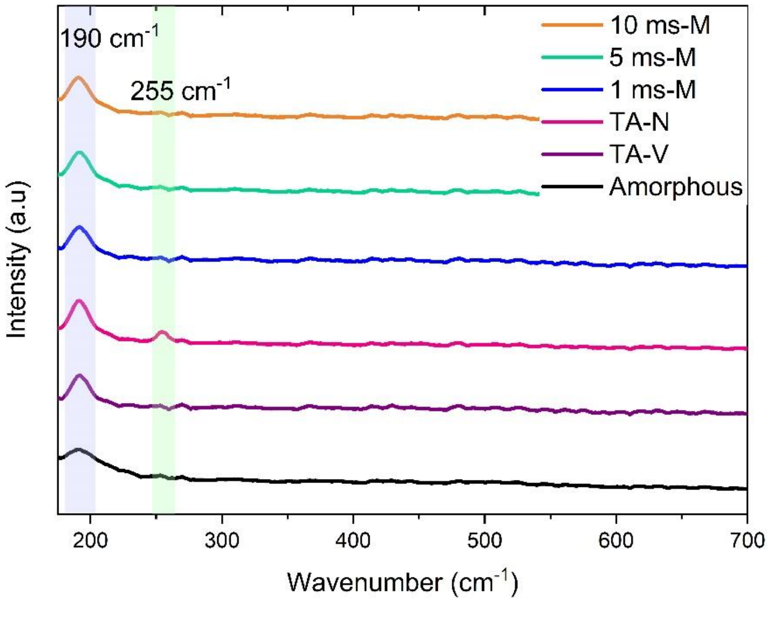

Figure S8. Raman spectra of amorphous, TA-V, TA-N, 1 ms-M, 5 ms-M, and 10 ms-M Sb_2Se_3 films as indicated in Table 1.

XPS spectra of 300 nm amorphous Sb_2Se_3 and PC Sb_2Se_3 films (cured with 0.5 ms, 450V, 0.5 Hz, 3 pulses, 1.4 J cm^{-2} per pulse) are shown in Figures S9 and S10, respectively. While these samples were fabricated under different conditions, the results of the analysis shown in Figures S9 and S10 show a similar evolution of surface oxide and elemental selenium as previous reports on CSS-deposited samples[4] and analysis of Sb_2Se_3 surfaces in general.[5] The angle-

dependant analysis (Figure S9 and S10) shows these species are surface localised, and given the prominence of Sb-Se and Se-Sb peaks, even at high angles (more surface sensitivity), only the upper nanometers of the sample surface is affected. This also explains the lack of oxide signal from EDS or Raman analysis. The Se 3d plots show distinct differences between the envelope and data, which is attributed to an underlying energy loss feature (plasmon) peak originating from the Sb 4d peaks (~33 eV) and previously reported.[5]

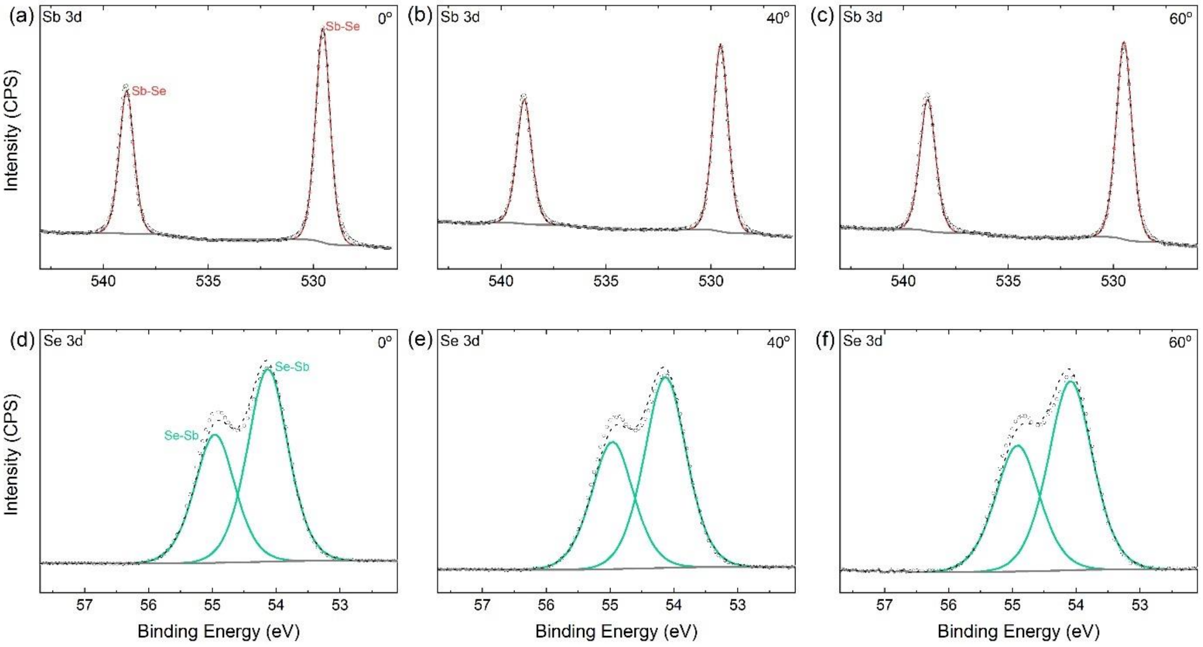

Figure S9. XPS spectra of amorphous Sb_2Se_3 varied substrate angle from 0° to 60°. The Sb 3d orbital region is shown in the top row, with the Se 3d orbital region shown in the bottom row.

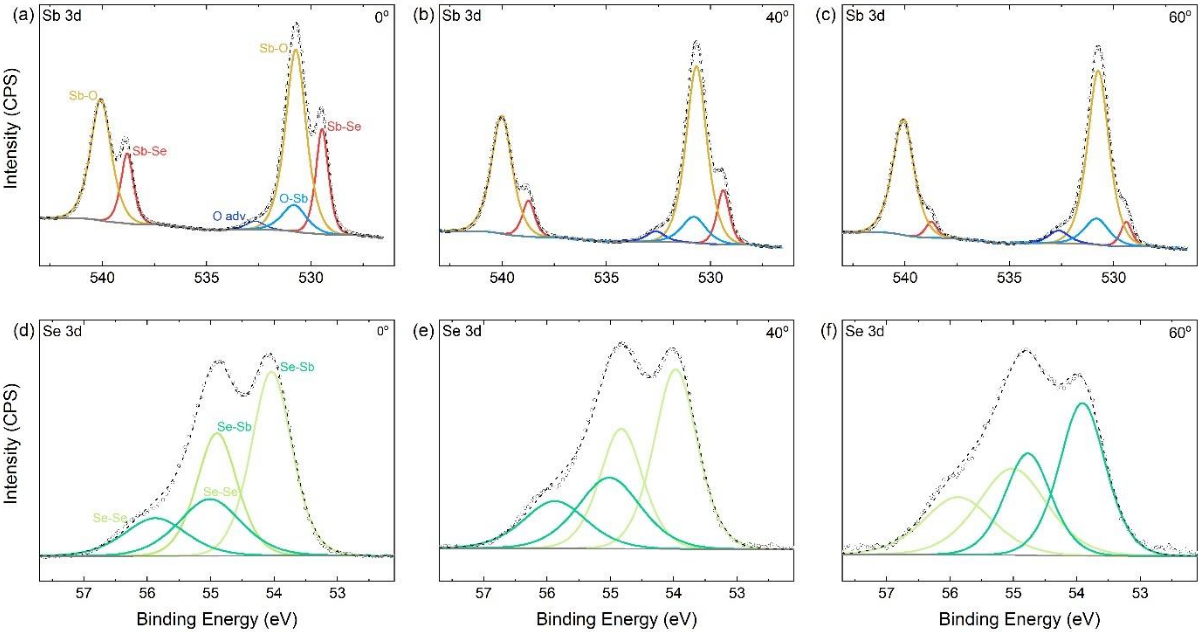

Figure S10. XPS spectra of the PC cured Sb_2Se_3 at varied substrate angles from 0° to 60°. The Sb 3d orbital region is shown in the top row, with the Se 3d orbital region shown in the bottom row.

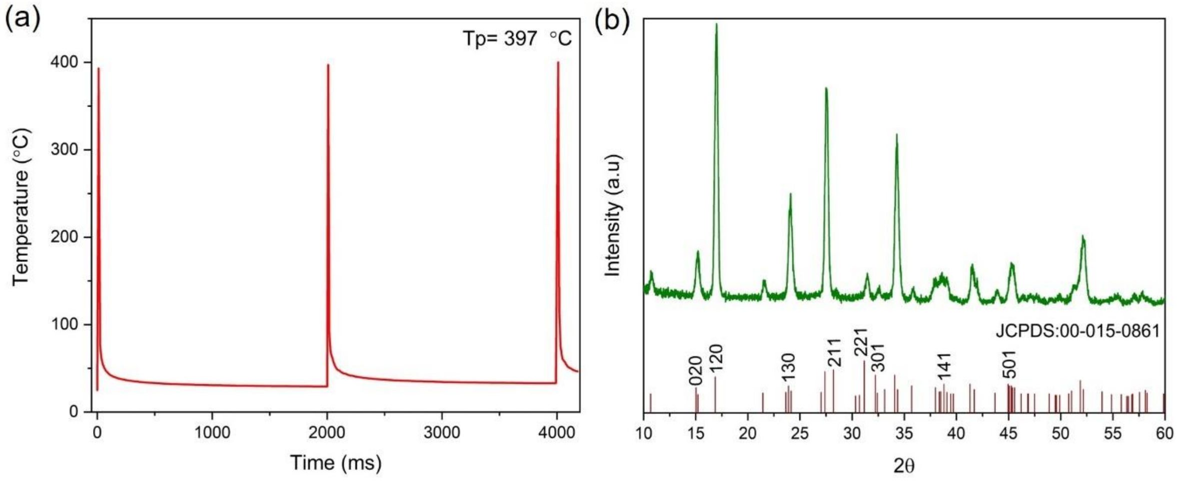

Figure S11. (a) Simulated temperature response of 300 nm Sb_2Se_3 on Glass/FTO/TiO_2. (b) XRD pattern of PC 300 nm Sb_2Se_3 on Glass/FTO/TiO_2 (0.5 ms, 450V, 0.5 Hz, 3 pulses, 1.4 J cm^{-2} per pulse).

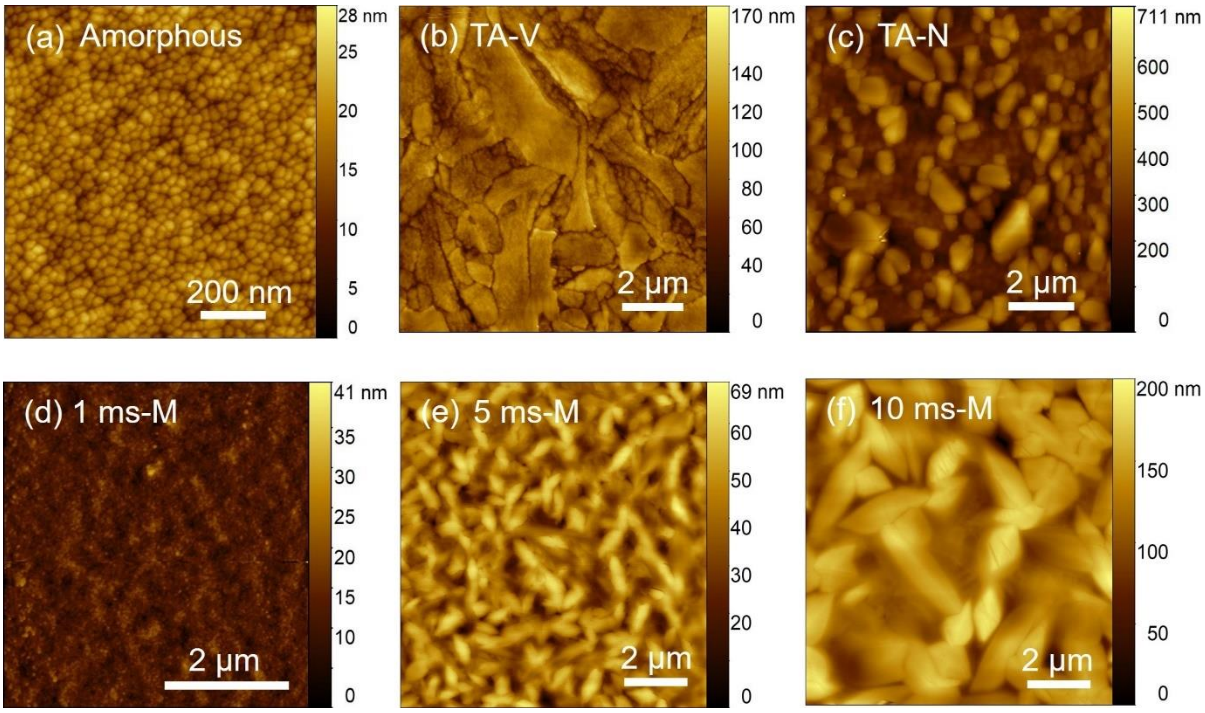

Figure S12. AFM surface images of Sb_2Se_3 films (a) Amorphous, (b) TA-V, (c) TA-N, (d) 1 ms-M, (e) 5 ms-M, and (f)10 ms-M as indicated (Table 1).

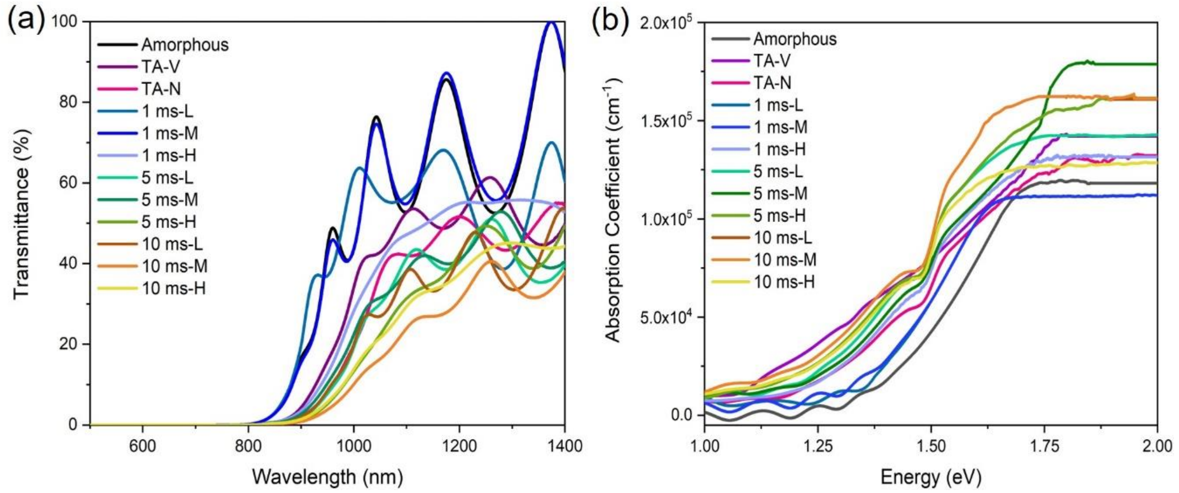

Figure S13. (a) Transmittance spectra. (b) Absorption coefficient versus photon energy graph for amorphous Sb_2Se_3 and post-annealed Sb_2Se_3 with conditions mentioned in Table 1.

Table S2. Optical properties of Sb_2Se_3 films mentioned in Table 1.

Sample	Absorption Coefficient * ($\times 10^5$ cm^{-1})	Band gap (eV)	Urbach Energy (meV)
Amorphous	1.18	1.63	187.6
TA-V	1.42	1.18	205.0
TA-N	1.32	1.25	143.7
1 ms-M	1.12	1.40	132.4
5 ms-M	1.79	1.32	162.1
10 ms-M	1.62	1.27	193.4

*Absorption coefficients were calculated at wavelength of 550 nm.

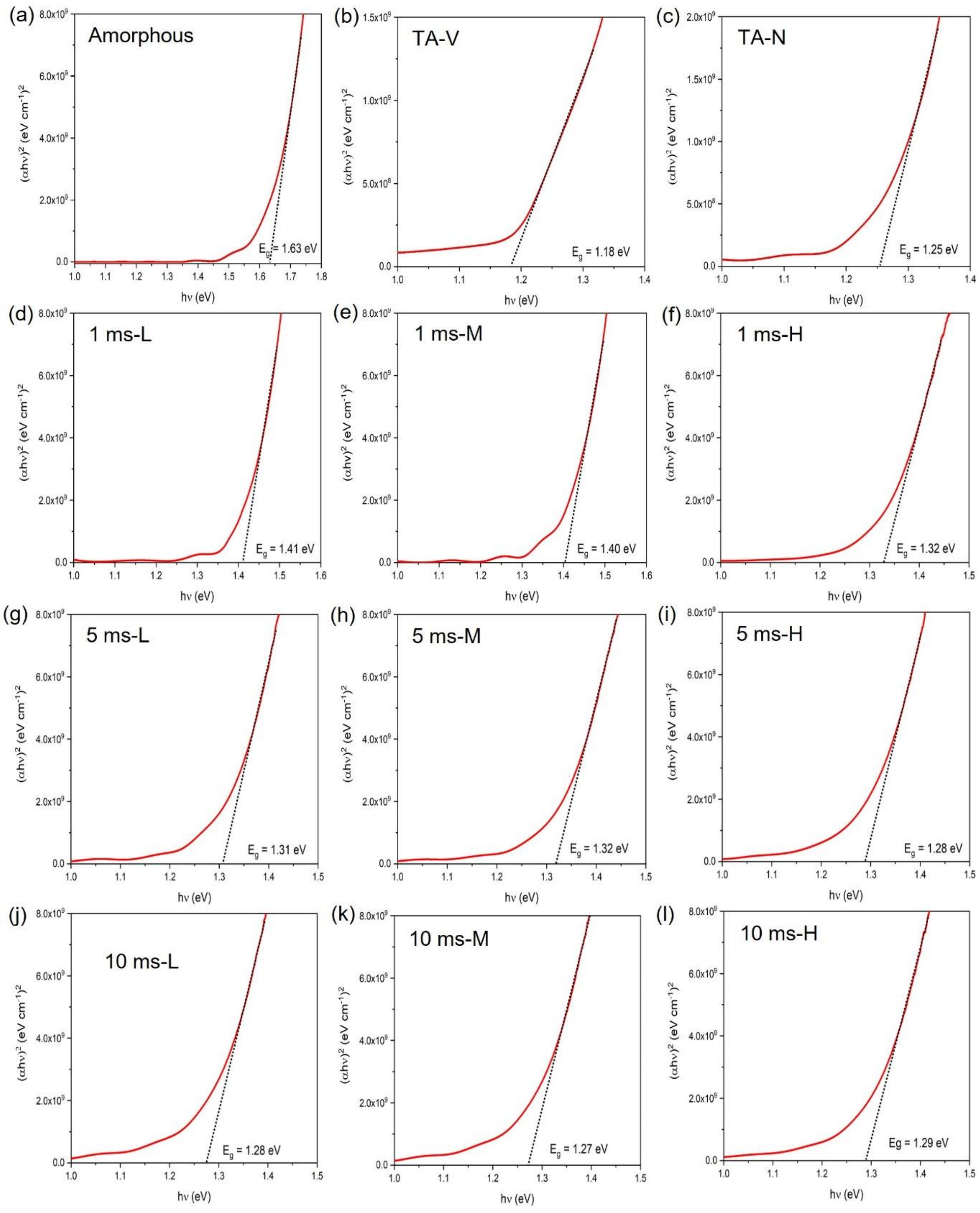

Figure S14. Optical band gaps of Amorphous Sb_2Se_3 and post-annealed Sb_2Se_3 with conditions mentioned in Table 1.

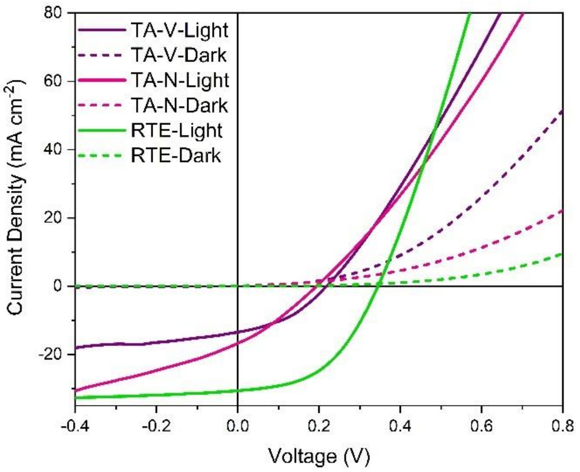

Sample	J_{SC} (mA cm^{-2})	V_{OC} (mV)	FF (%)	PCE (%)
TA-N	16.72	193.80	29.79	0.97
TA-V	13.46	218.50	38.79	1.14
RTE	30.56	338.56	48.84	5.00

Figure S15. J-V curves of solar cell fabricated with RTE and TA Sb_2Se_3.

RTE Device Fabrication

All devices were deposited on FTO-coated glass substrates (FTO: TEC15 Sigma Aldrich) that had been cleaned using sequential ultrasonic baths of DI water, acetone, and IPA, followed by 15-minute UV-ozone (Ossila) treatment. Then, the TiO_2 buffer layer was deposited by spin coating two doses of 0.30 M titanium isopropoxide solutions in ethanol at 3000 rpm for 30 s and dried at 120 ℃ for 10 min in the glove box after each deposition step. To obtain high quality TiO_2 buffer layers, the as-deposited TiO_2 was annealed in air at 450 °C for 30 min. Sb_2Se_3 thin films were deposited by RTE (OTF-1200X-RTP, MTI, California, USA). When the pressure was reduced below 10 mtorr, the film deposition was initiated. Sb_2Se_3 thin films were deposited on the FTO/TiO_2 substrates at 470 ℃ for 600 s, then the heating was turned off and the films were allowed to cool down to ~50 °C naturally before being removed from the evaporator.

References

1. Aryana, K.; Kim, H. J.; Islam, M. R.; Hong, N.; Popescu, C.-C.; Makarem, S.; Gu, T.; Hu, J.; Hopkins, P. E., Optical and thermal properties of Ge 2 Sb 2 Te 5, Sb 2 Se 3, and Sb 2 S 3 for reconfigurable photonic devices. *Optical Materials Express* **2023,** *13* (11), 3277-3286.
2. Mavlonov, A.; Razykov, T.; Raziq, F.; Gan, J.; Chantana, J.; Kawano, Y.; Nishimura, T.; Wei, H.; Zakutayev, A.; Minemoto, T., A review of Sb2Se3 photovoltaic absorber materials and thin-film solar cells. *Solar Energy* **2020,** *201*, 227-246.
3. Pashinkin, A.; Malkova, A.; Mikhailova, M., The heat capacity of solid antimony selenide. *Russian Journal of Physical Chemistry A, Focus on Chemistry* **2008,** *82*, 1035-1036.
4. Fleck, N.; Hutter, O. S.; Phillips, L. J.; Shiel, H.; Hobson, T. D.; Dhanak, V. R.; Veal, T. D.; Jäckel, F.; Durose, K.; Major, J. D., How oxygen exposure improves the back contact and performance of antimony selenide solar cells. *ACS applied materials & interfaces* **2020,** *12* (47), 52595-52602.
5. Don, C. H.; Shiel, H.; Hobson, T. D.; Savory, C. N.; Swallow, J. E.; Smiles, M. J.; Jones, L. A.; Featherstone, T. J.; Thakur, P. K.; Lee, T.-L., Sb 5s 2 lone pairs and band

alignment of Sb 2 Se 3: a photoemission and density functional theory study. *Journal of Materials Chemistry C* **2020,** *8* (36), 12615-12622.
